# Three New Isoprenylated Flavonoids from the Root Bark of *Morus alba*

**DOI:** 10.3390/molecules21091112

**Published:** 2016-08-24

**Authors:** Jae-Woo Jung, Ji-Hae Park, Yeong-Geun Lee, Kyeong-Hwa Seo, Eun-Ji Oh, Dae-Young Lee, Dong-Wook Lim, Daeseok Han, Nam-In Baek

**Affiliations:** 1 Graduate School of Biotechnology and Oriental Medicine Biotechnology, Kyung Hee University, Yongin 17104, Korea; jaewoo4848@naver.com (J.-W.J.); pjh3411@kbsi.re.kr (J.-H.P.); lyg629@nate.com (Y.-G.L.); JK3172@nate.com (E.-J.O.); 2 Natural Medicine Research Center, KRIBB, Chengju 28116, Korea; khseo@kribb.re.kr; 3 Department of Herbal Crop Research, National Institute of Horticultural and Herbal Science, RDA, Eumseong 27709, Korea; dylee0809@korea.kr; 4 Division of Metabolism and Functionality Research, Korea Food Research Institute, Sungnam 463-746, Korea; neodw4015@kfri.re.kr (D.-W.L.); imissu@kfri.re.kr (D.H.)

**Keywords:** isoprenylated flavonoids, *Morus alba*, root bark, sanggenon U, sanggenon V, sanggenon W

## Abstract

Phytochemical investigation of the root bark of *Morus alba* has led to the isolation and identification of three new isoprenylated flavonoids, namely sanggenon U (**1**), sanggenon V (**2**), and sanggenon W (**3**), along with four known isoprenylated flavonoids: euchrenone a_7_ (**4**), sanggenon J (**5**), kuwanon E (**6**), and kuwanon S (**7**). All compounds were isolated by repeated silica gel (SiO_2_), octadecyl SiO_2_ (ODS), and Sephadex LH-20 open column chromatography. The structure of the compounds were determined based on spectroscopic analyses, including nuclear magnetic resonance (NMR), mass spectrometry (MS), circular dichroism (CD), and infrared (IR). In addition, compounds **1**–**4** were isolated for the first time from the root bark of *M. alba* in this study.

## 1. Introduction

An isoprenylated flavonoid is formed by attachment of various prenyl moieties to a flavanone, flavone, flavanonol, flavonol, isoflavone, or chalcone. Barron et al. [1] proposed more than 600 structurally diverse prenylated flavonoids. Prenylated flavonoids are hybrid products composed of a flavonoid core usually attached to either 5-carbon or 10-carbon prenyl moieties derived from isoprenoid metabolism. Prenylated flavonoids are widely distributed in the Leguminosae, Moraceae, Euphorbiaceae, Guttiferae, and Umbelliferae plant families [1] and exhibit a variety of biological activities including anticancer [2,3] and the regulation of blood pressure via inhibition of NO production [4]. Especially, the addition of an isoprenoid moiety confers higher activity to the prenylated flavonoid molecule than in the parent flavonoid compound from the pharmacological point of view [5]. The proposed reason for the enhanced biological activity of prenylated flavonoid is that the attachment of the prenyl moiety to the flavonoid core increases the lipophilicity and the membrane permeability of the compound. In previous reports, several isoprenylated flavonoids, sanggenols, sanggenons, and kuwanons, were isolated from *Morus alba* L. in the family of Moraceae [6].

The mulberry tree (*Morus alba* L., Moraceae) is native to Thailand, and is widely cultivated in China, Korea, and Japan. Mulberry leaves, as the indispensable food of silkworms, are economically important sources for sericulture in East Asia [7]. Most parts of this tree has been widely used for a variety of medicinal purposes. The root bark, named “Sang-Bai-Pi”, has been used for treating diabetics, relieving asthma, and protecting the liver [8]. Previously reported mulberry root bark compounds include isoprenylated flavonoids, Diels-Alder type adducts, triterpenoids, coumarins, benzofurans, and stilbenes [9,10,11,12,13]. These compounds are reported to show anti-oxidant, anti-inflammatory, anti-hepatitis B virus, anti-cancer, and anti-microbial activities [14,15,16,17]. The EtOAc soluble fractions of mulberry root bark recently revealed antidepressant effects in vivo [18] as well as isolation of a new hydroxyl fatty acid [19]. Therefore, isolation of active compounds from the root bark of *M. alba* was carried out. This paper describes the procedure for the isolation of three new and four known isoprenylated flavonoids through solvent extraction, solvent fractionation, and open column chromatography as well the identification of the chemical structure of the compounds on the basis of spectroscopic analyses such as NMR, IR, and MS experiments.

## 2. Results and Discussion

**Figure 1:**
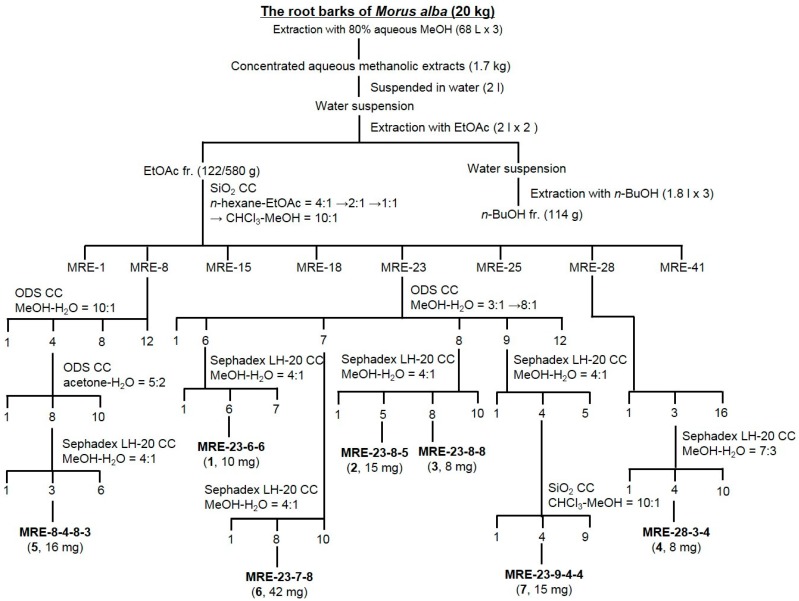
Extraction, fractionation, and isolation scheme of isoprenylated flavonoids from the root bark of *Morus alba*. SiO_2_: silica gel; CC: column chromatography; ODS: octadecyl silica gel; MRE: EtOAc fraction of *Morus alba* root bark.

Repeated open column chromatography (SiO_2_, ODS, and Sephadex LH-20 resins) of the EtOAc fraction from the *M. alba* root bark resulted in the isolation of seven isoprenylated flavonoids, including three new compounds, named sanggenon U (**1**), sanggenon V (**2**), and sanggenon W (**3**), and four known compounds (**4**–**7**, Figure 1). The chemical structures of the isolated isoprenylated flavonoids were determined based on the analyses of 1D-NMR (^1^H and ^13^C) and 2D-NMR (DEPT, HSQC, HMBC, and COSY), MS, CD, and IR spectroscopic data. The known compounds were finally identified to be euchrenone a_7_ (**4**), sanggenon J (**5**), kuwanon E (**6**), and kuwanon S (**7**) by comparison of the spectroscopic data with those previously reported in the literature [20,21,22,23]. ^1^H-NMR, and ^13^C-NMR spectra of three new compounds **1**, **2** and **3** are available on the Supplementary Materials.

**Figure 2:**
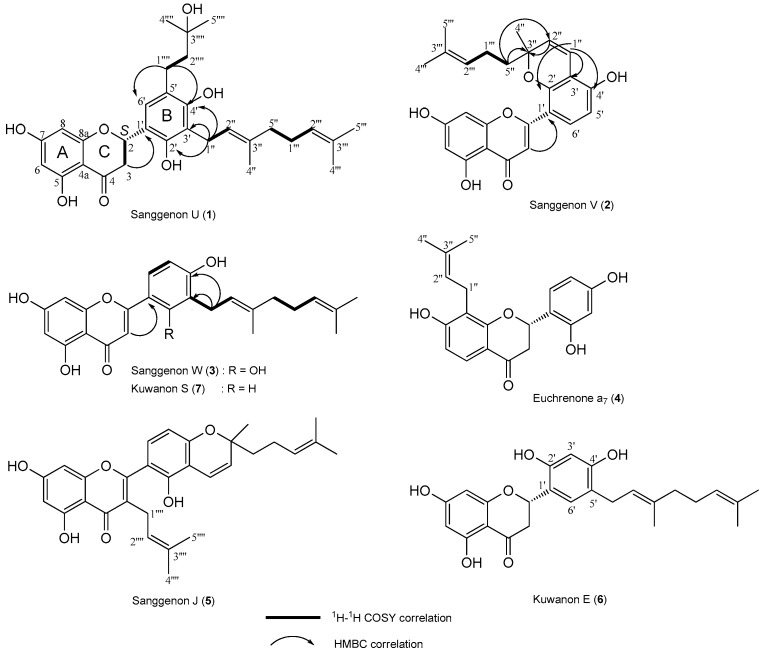
Chemical structures of compounds **1**–**7** from the root bark of *Morus alba* and key ^1^H-^1^H COSY and HMBC correlations for compounds **1**–**3**.

Compound **1** was isolated as a yellow amorphous powder and showed characteristic UV absorptions at 254 and 365 nm and also a yellow color on the TLC plate when sprayed with 10% sulfuric acid and heating. The molecular weight was determined to be 510 from the molecular ion peak *m*/*z* 510 [M]^+^ in the EI/MS spectrum, and a molecular formula of C_30_H_38_O_7_ according to the high-resolved molecular ion peak *m*/*z* 510.2616 [M]^+^ (calcd for C_30_H_38_O_7_, 510.2618,) in the HR/EI/MS. IR absorbance bands of hydroxyl (3373 cm^−1^), carbonyl (1662 cm^−1^), and aromatic (1608, 1577 cm^−1^) groups were observed. In the ^1^H-NMR spectrum, one aromatic signal at δ_H_ 7.03 (1H, s, H-6′) owing to a pentasubstituted benzene ring B and two aromatic signals at δ_H_ 5.90 (1H, d, *J* = 2.0 Hz, H-6) and 5.86 (1H, d, *J* = 2.0 Hz, H-8) due to a 1,2,3,5-tetrasubstituted benzene ring A were observed. In addition, the oxygenated methine signal at δ_H_ 5.64 (1H, dd, *J* = 12.8, 2.8 Hz, H-2), the methylene signals at δ_H_ 3.08 (1H, dd, *J* = 17.2, 12.8 Hz, H-3a) and 2.69 (1H, dd, *J* = 17.2, 2.8 Hz, H-3b) indicated the AMX system typical of a flavanone ring C. The geranyl moiety proton signals such as two olefinic methines at δ_H_ 5.18 (1H, t, *J* = 6.8 Hz, H-2′′) and 5.06 (1H, t, *J* = 6.8 Hz, H-2′′′), three methylenes at δ_H_ 3.41 (2H, d, *J* = 6.8 Hz, H-1′′), 2.06 (2H, dt, *J* = 6.8, 6.8 Hz, H-1′′′), and 1.98 (2H, d, *J* = 6.8 Hz, H-5′′), and three methyls at δ_H_ 1.78 (3H, s, H-4′′), 1.62 (3H, s, H-4′′′), and 1.56 (3H, s, H-5′′′) were observed. Moreover, two methylenes at δ_H_ 2.63 (2H, m, H-1′′′′) and 1.71 (2H, m, H-2′′′′), and two methyls at δ_H_ 1.24 (3H, s, H-4′′′′) and 1.24 (3H, s, H-5′′′′) proton signals indicated the presence of a prenyl moiety. The abovementioned evidence suggested that compound **1** was a tetrahydroxyflavanone compound with a geranyl and a prenyl group. The ^13^C-NMR spectrum showed 30 carbon signals. The tetrahydroxy flavanone moiety showed one ketone signal at δ_C_ 198.25 (C-4), five oxygenated olefinic quaternary signals at δ_C_ 168.85 (C-7), 165.52 (C-8a), 165.22 (C-5), 154.46 (C-4′), and 151.77 (C-2′), four olefinic quaternary signals at δ_C_ 123.40 (C-5′), 119.62 (C-1′), 118.74 (C-3′), and 103.23 (C-4a), three olefinic methine signals at δ_C_ 125.93 (C-6′), 97.18 (C-8), and 96.40 (C-6), one oxygenated methine signal at δ_C_ 76.72 (C-2), and one methylene signal at δ_C_ 43.35 (C-3). Moreover, two olefinic quaternary signals at δ_C_ 136.44 (C-3′′) and 132.24 (C-3′′′), two olefinic methine signals at δ_C_ 125.37 (C-2′′′) and 123.99 (C-2′′), three methylene signals at δ_C_ 40.90 (C-5′′), 27.63 (C-1′′′), and 23.77 (C-1′′), and three methyl signals at δ_C_ 25.89 (C-4′′′), 17.74 (C-5′′′), and 16.36 (C-4′′) derived from a geranyl moiety were observed. The prenyl moiety carbon signals, one oxygenated quaternary signal at δ_C_ 71.58 (C-3′′′′), two methylene signals at δ_C_ 45.05 (C-2′′′′) and 25.97 (C-1′′′′), and two methyl signals at δ_C_ 29.31 (C-4′′′′) and 29.31 (C-5′′′′) were observed. The flavanone structure and the location of the geranyl and prenyl groups were determined on the basis of the COSY and HMBC NMR experiments (Figure 2). In the COSY spectrum, the oxygenated methine proton signal at δ_H_ 5.64 (H-2) showed cross peaks with the methylene proton signals at δ_H_ 3.08 (H-3a) and 2.69 (H-3b), confirming the flavanone ring C structure. The two methylene proton signals at δ_H_ 3.41 (H-1′′) and 2.63 (H-1′′′′) were correlated with the olefinic methine proton signal at δ_H_ 5.18 (H-2′′) and the methylene proton signal at δ_H_ 1.71 (H-2′′′′), respectively. The methylene proton signal at δ_H_ 2.06 (H-1′′′) showed cross peaks with the olefinic methine proton signal at δ_H_ 5.06 (H-2′′′) and the methylene proton signal at δ_H_ 1.98 (H-5′′). In the HMBC spectrum, the allyl methylene proton signal of the geranyl moiety at δ_H_ 3.41 (H-1′′) showed cross peaks with the oxygenated olefinic quaternary carbon signals at δ_C_ 154.46 (C-4′) and 151.77 (C-2′), and the olefinic quaternary carbon signal at δ_C_ 118.74 (C-3′) indicating the geranyl moiety was linked to C-3′ in the flavanone B ring. The correlation of the methylene proton signal of the prenyl moiety at δ_H_ 2.63 (H-1′′′′) with the oxygenated olefinic quaternary carbon signal at δ_C_ 154.46 (C-4′), the olefinic methine carbon signal at δ_C_ 125.93 (C-6′), and olefinic quaternary carbon signal at δ_C_ 123.40 (C-5′) indicated the prenyl moiety was linked to C-5′ in the flavanone B ring. The absolute configuration of C-2 was determined to be (*S*)- from positive Cotton effect at 328 nm and the negative Cotton effect at 279 nm in the CD spectrum [24]. Taken together, compound **1** was determined to be (2*S*)-5,7,2′,4′-tetrahydroxy-3′-(3,7-dimethyl-octa-2,6-dienyl)-5′-(3-hydroxy-3-methylbutyl)flavanone, a new isoprenylated flavonoid, which was named sanggenon U.

Compound **2** was isolated as a yellow amorphous powder and showed characteristic UV absorptions at 254 and 365 nm in addition to a yellow color on the TLC plate when sprayed with 10% sulfuric acid and heating. The molecular weight was determined to be 420 from the molecular ion peak *m*/*z* 420 [M]^+^ in the EI/MS spectrum, and a molecular formula of C_25_H_24_O_6_ according to the high-resolved molecular ion peak *m*/*z* 420.1572 [M]^+^ (calcd for C_25_H_24_O_6_, 420.1573) in the HR/EI/MS. IR absorbance bands of hydroxyl (3382 cm^−1^), conjugated ketone (1666 cm^−1^), and aromatic (1598, 1545 cm^−1^) groups were observed. The ^1^H-NMR spectrum exhibited two olefinic methine signals of a 1,2,3,4-tetrasubstituted benzene ring B at δ_H_ 7.62 (1H, d, *J* = 8.4 Hz, H-6′) and 6.49 (1H, d, *J* = 8.4 Hz, H-5′) and two olefinic methine signals of a typical meta-coupled pattern due to a 1,2,3,5-tetrasubstituted benzene ring A at δ_H_ 6.38 (1H, br.s, H-8) and 6.17 (1H, br.s, H-6). The olefinic methine signal at δ_H_ 7.04 (1H, s, H-3) indicated the characteristic of flavone ring C structure. In addition, the olefinic methine signals at δ_H_ 6.72 (1H, d, *J* = 10.0 Hz, H-1′′) and 5.63 (1H, d, *J* = 10.0 Hz, H-2′′) of a prenyl moiety indicated the formation of a pyran ring through cyclization between the oxygenated olefinic quaternary carbon at δ_C_ 110.84 (C-3′) and the oxygenated quaternary carbon at δ_C_ 81.37 (C-3′′). This cyclization, a type of isoprene side chain with an *ortho*-phenolic hydroxyl, leads to pyrano or furano derivatives [1]. The other prenyl moiety showed one olefinic methine at δ_H_ 5.08 (1H, t, *J* = 6.8 Hz, H-2′′′), one methylene at δ_H_ 2.08 (2H, m, H-1′′′), and two methyls at δ_H_ 1.58 (3H, s, H-4′′′) and 1.47 (3H, s, H-5′′′) proton signals. The abovementioned evidence suggested that compound **2** was a tetrahydroxyflavone compound with a pyran ring type of prenyl substituent and a prenyl group. The ^13^C-NMR spectrum showed 25 carbon signals. The tetrahydroxyflavone moiety showed one conjugated ketone signal at δ_C_ 184.20 (C-4), six oxygenated olefinic quaternary signals at δ_C_ 166.00 (C-7), 163.49 (C-2), 163.06 (C-5), 159.42 (C-8a), 158.05 (C-4′), and 155.25 (C-2′), three olefinic quaternary signals at δ_C_ 111.53 (C-1′), 110.84 (C-3′), and 105.12 (C-4a), five olefinic methine signals at δ_C_ 111.53 (C-6′), 110.84 (C-3′), 108.59 (C-3), 99.92 (C-6), and 94.86 (C-8) were observed. In addition, two olefinic methines at δ_C_ 128.46 (C-2′′) and 118.19 (C-1′′), one oxygenated quaternary at δ_C_ 81.37 (C-3′′), one methylene at δ_C_ 42.17 (C-5′′), and one methyl at δ_C_ 26.89 (C-4′′) carbon signals were observed as the signals of a pyran ring type of a prenyl moiety. The other prenyl moiety showed one olefinic quaternary at δ_C_ 132.64 (C-3′′′), one olefinic methine at δ_C_ 125.04 (C-2′′′), one methylene at δ_C_ 23.98 (C-1′′′), and two methyls at δ_C_ 25.79 (C-4′′′) and 17.60 (C-5′′′) carbon signals. The flavone structure and the location of the prenyl groups were proved by COSY and HMBC NMR experiments (Figure 2). The HMBC experiment suggested the ether linkage of the pyran ring could be formed at either C-2′ or C-4′ of ring B. Accordingly, to determine the exact position of the ether linkage in ring B, UV absorption shift experiment was carried out, which confirms the presence of the free hydroxy at C-4′ of falvone B ring. Compound **2** was dissolved in MeOH/NaOCH_3_ resulted in red shift from 259 to 281 nm and from 353 to 398 nm, respectively, owing to the 4′-OH. Therefore, the ether linkage of the pyran ring was revealed to be at OH-2′ of B ring, which was confirmed by the key correlations in the HMBC spectrum (Figure 2), that is, H-5′′/C-3′′ and C-2′′; H-2′′/C-3′′; H-1′′/C-2′, C-3′, and C-4′. Taken together, compound **2** was determined to be 5,7,2′,4′-tetrahydroxy-2′,3′-(2-methyl-2-methylenechromeno)-5′′-(3-methylbut-2-enyl)flavone, a new isoprenylated flavonoid, that was named sanggenon V.

Compound **3** was isolated as a yellow amorphous powder and showed characteristic UV absorptions and yellow color on a TLC plate when sprayed with 10% sulfuric acid and heated. The molecular weight was determined to be 422 from the molecular ion peak *m*/*z* 422 [M]^+^ in the EI/MS spectrum, and the molecular formula of C_25_H_26_O_6_ according to the high-resolved molecular ion peak *m*/*z* 422.1726 [M]^+^ (calcd for C_25_H_26_O_6_, 422.1729) in the HR/EI/MS. The IR absorbance bands of hydroxyl (3376 cm^−1^), conjugated ketone (1659 cm^−1^), and aromatic (1588, 1541 cm^−1^) groups were observed. The ^1^H-NMR and ^13^C-NMR data of compound **3** were quite similar to those of **1**, except for the respective replacements of the oxygenated methine signal (δ_H_ 5.64, δ_C_ 76.72) and the methylene signal (δ_H_ 3.08, 2.69, δ_C_ 43.35) in the ring C of **1** by the oxygenated olefinic quaternary signal (δ_C_ 165.74) and the olefinic methine signal (δ_H_ 6.86, δ_C_ 108.38) in **3**, and disappearance of the prenyl moiety signals at C-5′ position. The carbon chemical shift of ketone signal at C-4 (δ_C_ 184.16) was shifted upfield by 14.09 ppm, comparing with the carbon chemical shift of **1** (δ_C_ 198.25) due to the conjugation effects, indicating a double bond to be between C-2 and C-3. Those findings indicated compound **3** was a tetrahydroxyflavone compound with a geranyl moiety. The tetrahydroxyflavone structure and the location of the geranyl moiety in **3** was confirmed based on the COSY and HMBC experiments (Figure 2). Thus, the structure of compound **3** was determined to be 5,7,2′,4′-tetrahydroxy-3′-(3,7-dimethyl-octa-2,6-dienyl)-flavone, a new isoprenylated flavonoid, and named sanggenon W.

## 3. Experimental Section

### 3.1. Plant Materials

The dried root bark of *Morus alba* L. (Moraceae) were supplied by the Korea Food Research Institute (Sungnam, Korea) in January 2012, and was identified by Professor Dae-Keun Kim, College of Pharmacy, Woosuk University, Jeonju, Korea. A voucher specimen (KHU-NPCL-201204) has been deposited at the Laboratory of Natural Products Chemistry, Kyung Hee University, Yongin, Korea.

### 3.2. General

Open column chromatography (CC) was carried out with a Kiesel gel 60 (Merck 60 Å, 70−230 mesh ASTM, Darmstadt, Germany), LiChroprep RP-18 (40~60 μm, Merck), and Sephadex LH-20 (Amersham Biosciences, Uppsala, Sweden). The thin layer chromatography (TLC) analysis was performed using Kieselgel 60 F_254_ and RP-18 F_254S_ (Merck) plates. The spots on TLC were detected using a UV lamp (Spectroline Model ENF-240 C/F, Spectronics Corporation, Westbury, NY, USA) and a 10% H_2_SO_4_ solution by spraying and heating. ^1^H- (400 MHz) and ^13^C-NMR (100 MHz, nuclear magnetic resonance) spectra were recorded on a Varian Unity Inova AS-400 FT-NMR spectrometer (Palo Alto, CA, USA). Infrared (IR) spectra were obtained from a Perkin-Elmer model 599B spectrophotometer (Waltham, MA, USA). Optical rotations were measured on a polarimeter (model P-1020, JASCO, Tokyo, Japan). Electronic ionization mass spectrometry (EI/MS) and fast atom bombardment mass spectrometry (FAB/MS) spectra were obtained using a JMSAX 700 (JEOL, Tokyo, Japan). Melting points were determined using Fisher-Johns melting point apparatus (Fisher Scientific, Miami, FL, USA) and not corrected. Circular dichroism (CD) spectra were obtained from a Chirascan Plus instrument (Applied Photophysics, Surrey, UK).

### 3.3. Extraction and Isolation

The fractionation and isolation procedure is described in Figure 1. The dried root bark of *M. alba* (10 kg) was extracted with 80% methanol (170 L) at room temperature for 24 h. The concentrated MeOH extract (1.7 kg) was suspended in 2 L of water and successively partitioned by increasing polarity gradients of ethyl acetate (EtOAc, 2 L × 2) and *n*-butyl alcohol (*n*-BuOH, 1.8 L × 3). The organic and aqueous layers were concentrated to produce the EtOAc fraction (MRE, 580 g), the *n*-BuOH fraction (MRB, 114 g), and the H_2_O fraction (MRW, 1006 g) residues, respectively. The MRE fraction (122 g) was subjected to a SiO_2_ CC (ϕ 12.5 × 15 cm) and eluted with *n*-hexane–EtOAc (4:1 → 2:1 → 1:1, 27 L of each) → CHCl_3_–MeOH (10:1, 27 L) with monitoring by TLC to obtain 41 fractions (MRE-1 to MRE-41). Fraction MRE-8 [848 mg, elution volume/total volume (Ve/Vt) 0.422−0.528] was subjected to an ODS CC (ϕ 6.5 × 12 cm) and eluted with MeOH–H_2_O (10:1, 1.8 L), yielding 12 fractions (MRE-8-1 to MRE-8-12). Subfraction MRE-8-4 (350 mg, Ve/Vt 0.106−0.119) was subjected to the ODS CC (ϕ 3.5 × 12 cm) and eluted with acetone–H_2_O (5:2, 1.1 L), yielding 10 fractions (MRE-8-4-1 to MRE-8-4-10). Subfraction MRE-8-4-8 (40 mg, Ve/Vt 0.710−0.747) was subjected to a Sephadex LH-20 CC (ϕ 1.5 × 60 cm) and eluted with MeOH–H_2_O (4:1, 0.5 L), yielding six fractions (MRE-8-4-8-1 to MRE-8-4-8-6) including a purified compound **5** at MRE-8-4-8-3 [16 mg, Ve/Vt 0.315-0.489, TLC (ODS) R_f_ 0.55, acetone–H_2_O = 5:1]. Fraction MRE-23 (848 mg, Ve/Vt 0.422−0.528) was applied to the ODS CC (ϕ 4.5 × 6 cm) and eluted with MeOH–H_2_O (3:1 → 8:1, 3 L of each), yielding 12 fractions (MRE-23-1 to MRE-23-12). Subfraction MRE-23-6 (60 mg, Ve/Vt 0.422−0.528) was subjected to the Sephadex LH-20 CC (ϕ 1.5 × 57 cm) and eluted with MeOH–H_2_O (4:1, 2 L), yielding seven fractions (MRE-23-6-1 to MRE-23-6-7) including a purified compound **1** at MRE-23-6-6 [10 mg, Ve/Vt 0.864–0.903, TLC (ODS) R_f_ 0.55, MeOH–H_2_O = 10:1]. Subfraction MRE-23-7 (400 mg, Ve/Vt 0.136−0.318) was subjected to the Sephadex LH-20 CC (ϕ 1.5 × 60 cm) and eluted with MeOH–H_2_O (4:1, 2.1 L), yielding 10 fractions (MRE-23-7-1 to MRE-23-7-10) including a purified compound **6** at MRE-23-7-8 [42 mg, Ve/Vt 0.760–0.833, TLC (ODS) R_f_ 0.44, MeOH–H_2_O = 6:1]. Subfraction MRE-23-8 (110 mg, Ve/Vt 0.319–0.506) was subjected to the Sephadex LH-20 CC (ϕ 2 × 67 cm) and eluted with MeOH–H_2_O (4:1, 2 L), yielding 10 fractions (MRE-23-8-1 to MRE-23-8-10) including a purified compound **2** at MRE-23-8-5 [15 mg, Ve/Vt 0.523–0.553, TLC (ODS) R_f_ 0.43, MeOH–H_2_O = 10:1] and a compound **3** at MRE-23-8-8 [8 mg, Ve/Vt 0.657–0.721, TLC (ODS) R_f_ 0.48, MeOH–H_2_O = 10:1]. Subfraction MRE-23-9 (120 mg, Ve/Vt 0.507–0.651) was subjected to the Sephadex LH-20 CC (ϕ 1.5 × 60 cm) and eluted with MeOH–H_2_O (4:1, 0.7 L), yielding five fractions (MRE-23-9-1 to MRE-23-9-5). Subfraction MRE-23-9-4 (52 mg, Ve/Vt 0.704–0.795) was subjected to the SiO_2_ CC (ϕ 2 × 15 cm) and eluted with CHCl_3_–MeOH (22:1, 0.4 L), yielding nine fractions (MRE-23-9-4-1 to MRE-23-9-4-9) including a purified compound **7** at MRE-23-9-4-4 [15 mg, Ve/Vt 0.505–0.555, TLC (ODS) R_f_ 0.41, MeOH–H_2_O = 12:1]. Fraction MRE-28 (2.2 g, Ve/Vt 0.595−0.670) was applied to the ODS CC (ϕ 7 × 4 cm) and eluted with MeOH–H_2_O (2:1→ 4:1 → 6:1, 2 L of each), yielding 16 fractions (MRE-28-1 to MRE-28-16). Subfraction MRE-28-3 (80 mg, Ve/Vt 0.021−0.032) was subjected to the Sephadex LH-20 CC (ϕ 1 × 60 cm) and eluted with MeOH–H_2_O (7:3, 1 L), yielding 10 fractions (MRE-28-3-1 to MRE-28-3-10) along with a purified compound **4** at MRE-28-3-4 (8 mg, Ve/Vt 0.214–0.283, TLC (ODS) R_f_ 0.55, MeOH–H_2_O = 2:1).

### 3.4. Spectroscopic Data

*Sanggenon U* (**1**). Yellow amorphous powder (MeOH). m.p. 155–160 °C. ${\left[\alpha \right]}_{D}^{25}$−39.7° (*c* 0.62, MeOH). IR_ν_ (CaF_2_ plate) 3373, 2924, 1662, 1608, 1577 cm^−1^. HR/EI/MS *m*/*z* 510.2616 [M]^+^ (calcd for C_30_H_38_O_7_, 510.2618). ^1^H-NMR (CD_3_OD) and ^13^C-NMR (CD_3_OD) data: see Table 1.

*Sanggenon V* (**2**). Yellow amorphous powder (MeOH). m.p. 145–150 °C. ${\left[\alpha \right]}_{D}^{25}$+2.1° (*c* 0.85, MeOH). IR_ν_ (CaF_2_ plate) 3382, 2944, 2863, 1666, 1598, 1545 cm^−1^. HR/EI/MS *m*/*z* 420.1572 [M]^+^ (calcd for C_25_H_24_O_6_, 420.1573). ^1^H-NMR (CD_3_OD) and ^13^C-NMR (CD_3_OD) data: see Table 1.

*Sanggenon W* (**3**). Yellow amorphous powder (MeOH). m.p. 100–105 °C. IR_ν_ (CaF_2_ plate) 3376, 2935, 2869, 1659, 1588, 1541 cm^−1^. HR/EI/MS *m*/*z* 422.1726 [M]^+^ (calcd for C_25_H_26_O_6_, 422.1729). ^1^H-NMR (CD_3_OD) and ^13^C-NMR (CD_3_OD) data: see Table 1.

*Euchrenone a_7_* (**4**). Yellow amorphous powder (MeOH). m.p. 110–115 °C. ${\left[\alpha \right]}_{D}^{25}$−34.8° (*c* 0.45, MeOH). IR_ν_ (CaF_2_ plate) 3310, 2922, 2887, 1667, 1601, 1518 cm^−1^. EI/MS *m*/*z* 340 [M]^+^. ^1^H-NMR (CD_3_OD, δ_H_) 7.58 (1H, d, *J* = 8.8 Hz, H-5), 7.27 (1H, d, *J* = 8.8 Hz, H-6′), 6.50 (1H, d, *J* = 8.8 Hz, H-6), 6.33 (1H, dd, *J* = 8.8, 2.0 Hz, H-5′), 6.32 (1H, d, *J* = 2.0 Hz, H-3′), 5.60 (1H, dd, *J* = 13.2, 2.8 Hz, H-2), 5.19 (1H, t, *J* = 6.8 Hz, H-2′′), 3.28 (2H, m, H-1′′), 2.92 (1H, dd, *J* = 17.2, 13.2 Hz, H-3a), 2.72 (1H, dd, *J* = 17.2, 2.8 Hz, H-3b), 1.75 (3H, s, H-4′′), 1.75 (3H, s, H-5′′); ^13^C-NMR (CD_3_OD, δ_C_) 195.03 (C-4), 163.84 (C-8a), 163.57 (C-7), 159.51 (C-4′), 156.65 (C-2′), 132.11 (C-3′′), 128.65 (C-6′), 126.74 (C-5), 123.38 (C-2′′), 118.57 (C-1′), 117.13 (C-8), 115.02 (C-4a), 110.69 (C-6), 107.64 (C-3′), 103.36 (C-5′), 76.29 (C-2), 44.03 (C-3), 25.98 (C-4′′), 22.99 (C-1′′), 17.95 (C-5′′).

*Sanggenon J* (**5**). Yellow amorphous powder (MeOH). m.p. 140–145 °C. ${\left[\alpha \right]}_{D}^{21}$−16.9° (*c* 0.03, CHCl_3_). EI/MS *m*/*z* 488 [M]^+^. IR (CaF_2_ plate, ν) 3374, 2928, 2892, 1661, 1605, 1514 cm^−1^. ^1^H-NMR (CD_3_OD, δ_H_) 6.97 (1H, d, *J* = 8.4 Hz, H-6′), 6.75 (1H, d, *J* = 10.0 Hz, H-1′′), 6.39 (1H, d, *J* = 8.4 Hz, H-5′), 6.26 (1H, d, *J* = 2.4 Hz, H-8), 6.17 (1H, d, *J* = 2.4 Hz, H-6), 5.63 (1H, d, *J* = 10.0 Hz, H-2′′), 5.09 (1H, t, *J* = 6.8 Hz, H-2′′′), 5.07 (1H, t, *J* = 6.8 Hz, H-2′′′′), 3.07 (2H, d, *J* = 6.8 Hz, H-1′′′′), 2.11 (2H, dt, *J* = 7.2, 6.8 Hz, H-1′′′), 1.71 (2H, t, *J* = 7.2 Hz, H-5′′), 1.65 (3H, s, H-5′′′), 1.58 (3H, s, H-4′′′), 1.56 (3H, s, H-5′′′′), 1.39 (3H, s, H-4′′), 1.33 (3H, s, H-4′′′′); ^13^C-NMR (CD_3_OD, δ_C_) 183.66 (C-4), 165.61 (C-7), 163.27 (C-5), 162.81 (C-2), 159.94 (C-8a), 157.36 (C-4′), 151.92 (C-2′), 132.83 (C-3′′′′), 132.49 (C-3′′′), 131.24 (C-6′), 129.35 (C-2′′), 125.24 (C-2′′′), 122.52 (C-2′′′′), 122.23 (C-3), 118.14 (C-1′′), 114.97 (C-1′), 111.72 (C-3′), 109.33 (C-5′), 105.50 (C-4a), 99.56 (C-6), 94.61 (C-8), 79.67 (C-3′′), 42.19 (C-5′′), 26.76 (C-4′′′′), 25.85 (C-5′′′), 25.85 (C-5′′′′), 24.68 (C-1′′′′), 23.75 (C-1′′), 17.69 (C-4′′′), 17.63 (C-4′′′′).

*Kuwanon E* (**6**). Yellow amorphous powder (MeOH). m.p. 120–125 °C. ${\left[\alpha \right]}_{D}^{25}$−0.25° (*c* 0.29, CH_3_OH). EI/MS *m*/*z* 424 [M]^+^. IR (CaF_2_ plate, ν) 3379, 2922, 2889, 1666, 1612, 1588 cm^−1^. ^1^H-NMR (CD_3_OD, δ_H_) 7.06 (1H, s, H-6′), 6.33 (1H, d, *J* = 8.4 Hz, H-3′), 5.89 (1H, d, *J* = 2.4 Hz, H-6), 5.86 (1H, d, *J* = 2.4 Hz, H-8), 5.58 (1H, dd, *J* = 13.2, 2.8 Hz, H-2), 5.28 (1H, t, *J* = 6.8 Hz, H-2′′), 5.08 (1H, t, *J* = 6.8 Hz, H-2′′′), 3.19 (2H, d, *J* = 7.2 Hz, H-1′′), 3.03 (1H, dd, *J* = 17.2, 13.2 Hz, H-3a), 2.68 (1H, dd, *J* = 17.2, 2.8 Hz, H-3b), 2.07 (2H, dt, *J* = 7.2, 6.8 Hz, H-1′′′), 2.00 (2H, t, *J* = 7.2 Hz, H-5′′), 1.66 (3H, s, H-4′′), 1.59 (3H, s, H-4′′′), 1.55 (3H, s, H-5′′′); ^13^C-NMR (CD_3_OD, δ_C_) 198.45 (C-4), 168.20 (C-8a), 165.43 (C-7), 165.35 (C-5), 156.95 (C-4′), 154.42 (C-2′), 136.44 (C-3′′), 132.11 (C-3′′′), 128.65 (C-6′), 125.41 (C-2′′′), 124.39 (C-2′′), 120.55 (C-5′), 117.29 (C-1′), 103.35 (C-3′), 103.34 (C-4a), 96.88 (C-6), 96.17 (C-8), 76.07 (C-2), 43.13 (C-3), 40.85 (C-5′′), 28.44 (C-1′′), 27.77 (C-1′′′), 25.86 (C-4′′′), 17.76 (C-5′′′), 16.18 (C-4′′).

*Kuwanon S* (**7**). Yellow amorphous powder (MeOH). m.p. 78–80 °C. EI/MS *m*/*z* 406 [M]^+^. IR (CaF_2_ plate, ν) 3359, 2913, 2884, 1671, 1601, 1584 cm^−1^. ^1^H-NMR (CD_3_OD, δ_H_) 7.59 (1H, d, *J* = 8.8 Hz, H-6′), 7.58 (1H, br.s, H-2′), 6.84 (1H, d, *J* = 8.8 Hz, H-5′), 6.44 (1H, s, H-3), 6.36 (1H, br.s, H-6), 6.15 (1H, br.s, H-8), 5.32 (1H, t, *J* = 6.8 Hz, H-2′′), 5.09 (1H, t, *J* = 6.8 Hz, H-2′′′), 3.31 (2H, d, *J* = 7.2 Hz, H-1′′), 2.11 (2H, dt, *J* = 7.2, 6.8 Hz, H-1′′′), 2.05 (2H, t, *J* = 7.2 Hz, H-5′′), 1.72 (3H, s, H-4′′), 1.59 (3H, s, H-4′′′), 1.56 (3H, s, H-5′′′); ^13^C-NMR (CD_3_OD, δ_C_) 183.72 (C-4), 166.44 (C-2), 166.03 (C-7), 163.11 (C-5), 160.51 (C-4′), 159.30 (C-8a), 137.64 (C-3′′), 132.11 (C-3′′′), 130.28 (C-3′), 128.79 (C-6′), 126.81 (C-2′′′), 125.26 (C-2′′′), 123.26 (C-2′′), 116.17 (C-5′), 105.21 (C-4a), 103.53 (C-3), 100.11 (C-6), 95.05 (C-8), 40.86 (C-5′′), 28.99 (C-1′′), 27.70 (C-1′′′), 25.85 (C-4′′′), 17.79 (C-5′′′), 16.27 (C-4′′).

## 4. Conclusions

Three new isoprenylated flavonoids **1**–**3** and four known ones **4**–**7** were isolated from the root bark of *M. alba*. Chemical structures of the isolated compounds were identified on the basis of NMR, MS, CD, and IR spectroscopic data. Compounds **1**–**4** were isolated for the first time from the root bark of *M. alba* in this study.

## Figures and Tables

**Table 1:** ^1^H- and ^13^C-NMR data (400 and 100 MHz, resp.; CD_3_OD) of isoprenylated flavonoids **1**–**3** from the root bark of *Morus alba*.

	Compound 1		Compound 2		Compound 3	
	δ_H_	δ_C_	δ_H_	δ_C_	δ_H_	δ_C_
2	5.64 (dd, *J* = 12.8, 2.8 Hz)	76.72		163.49		165.74
3	3.08 (dd, *J* = 17.2, 12.8 Hz)	43.35	7.04 (s)	108.59	6.86 (s)	108.38
	2.69 (dd, *J* = 17.2, 2.8 Hz)					
4		198.25		184.20		184.16
4a		103.23		105.12		105.13
5		165.22		163.06		163.09
6	5.90 (d, *J* = 2.0 Hz)	96.40	6.17 (s)	99.92	6.17 (s)	100.04
7		168.85		166.00		166.27
8	5.86 (d, *J* = 2.0 Hz)	97.18	6.38 (s)	94.86	6.39 (s)	95.08
8a		165.52		159.42		159.67
1′		119.62		111.53		112.62
2′		151.77		155.25		160.87
3′		118.74		110.84		118.10
4′		154.46		158.05		156.70
5′		123.40	6.49 (d, *J* = 8.4 Hz)	109.41	6.50 (d, *J* = 8.4 Hz)	109.20
6′	7.03 (s)	125.93	7.62 (d, *J* = 8.4 Hz)	129.80	7.46 (d, *J* = 8.4 Hz)	128.25
1′′	3.41 (d, *J* = 6.8 Hz)	23.77	6.72 (d, *J* = 10.0 Hz)	118.19	3.40 (d, *J* = 6.8 Hz)	23.05
2′′	5.18 (t, *J* = 6.8 Hz)	123.99	5.63 (d, *J* = 10.0 Hz)	128.46	5.20 (t, *J* = 6.8 Hz)	123.49
3′′		136.44		81.37		136.45
4′′	1.78 (s)	16.36	1.47 (s)	26.89	1.78 (s)	16.36
5′′	1.98 (d, *J* = 6.8 Hz)	40.90	1.79 (m)	42.17	1.97 (t, *J* = 6.8 Hz)	40.91
			1.69 (m)			
1′′′	2.06 (dt, *J* = 6.8, 6.8 Hz)	27.63	2.08 (m)	23.98	2.05 (dt, *J* = 6.8, 6.8 Hz)	27.67
2′′′	5.06 (t, *J* = 6.8 Hz )	125.37	5.08 (t, *J* = 6.8 Hz)	125.04	5.05 (t, *J* = 6.8 Hz)	125.38
3′′′		132.24		132.64		132.19
4′′′	1.62 (s)	25.89	1.58 (s)	25.79	1.59 (s)	25.85
5′′′	1.56 (s)	17.74	1.47 (s)	17.60	1.54 (s)	17.72
1′′′′	2.63 (m)	25.97				
2′′′′	1.71 (m)	45.05				
3′′′′		71.58				
4′′′′	1.24 (s)	29.31				
5′′′′	1.24 (s)	29.31				

## Supplementary Materials

^1^H-NMR, ^13^C-NMR, HMBC, and MS spectra are available as supporting data. Supplementary materials can be accessed at: http://www.mdpi.com/1420-3049/21/9/1112/s1.
