# Supplementary material for: Three New Isoprenylated Flavonoids from the Root Bark of Morus alba
Source: Molecules. 2016 Aug 24;21(9):1112. doi: 10.3390/molecules21091112 (PMC6272825; doi:10.3390/molecules21091112)
Supplement: Supplementary file 1 [file molecules-21-01112-s001.pdf]

Supplementary Materials: Three New Isoprenylated Flavonoids from the Root Bark of *Morus alba*

Jae-Woo Jung, Ji-Hae Park, Yeong-Geun Lee, Kyeong-Hwa Seo, Eun-Ji Oh, Dae-Young Lee, Dong-Wook Lim, Daeseok Han and Nam-In Baek

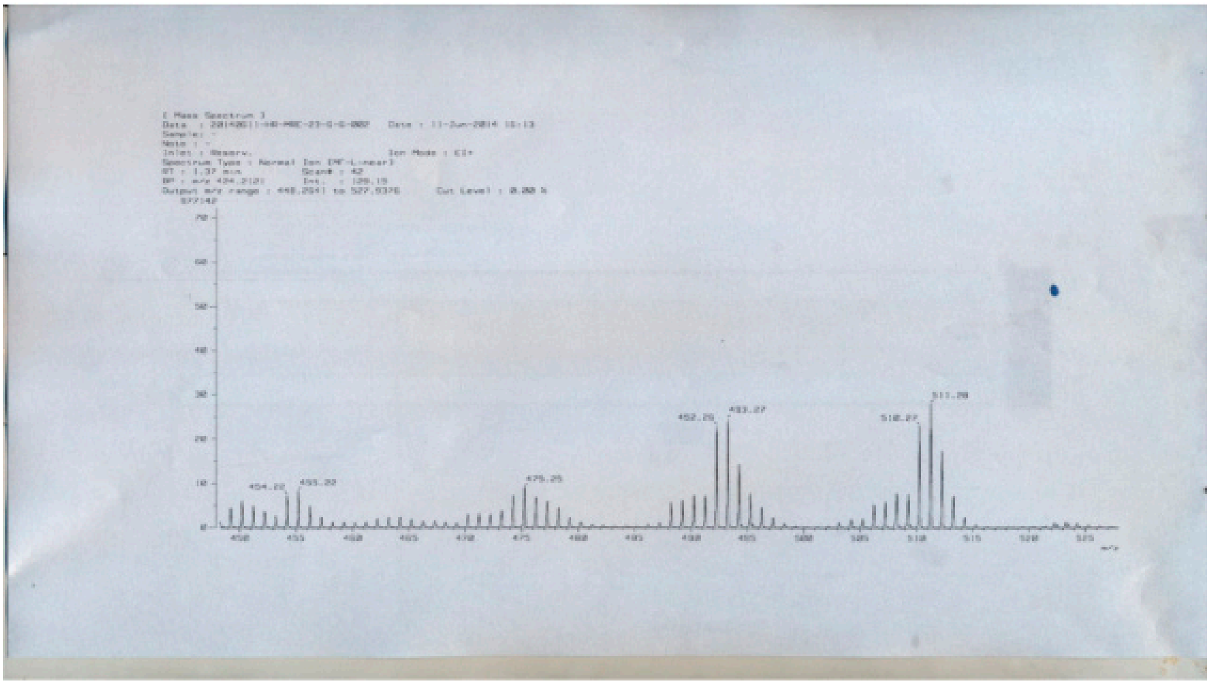

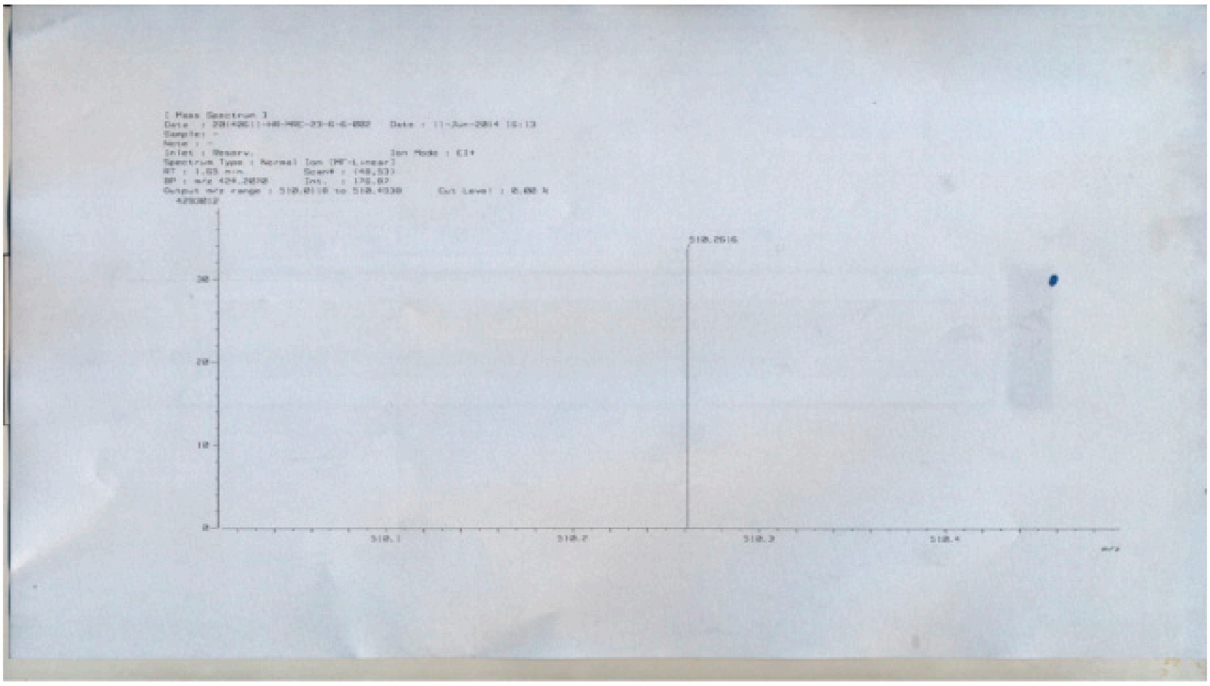

Figure S1. *Cont.*

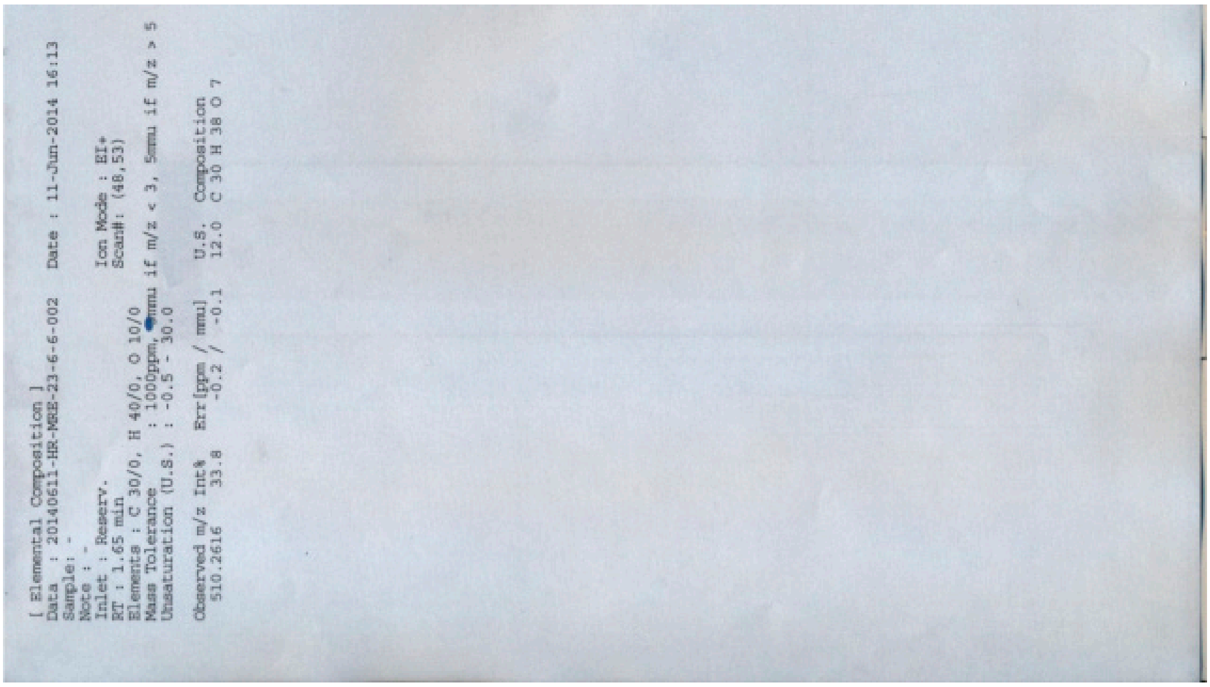

[Elemental Composition]
Data : 20140611-HR-MRE-23-6-6-002 Date : 11-Jun-2014 16:13
Sample: -
Note : -
Inlet : Reserv. Ion Mode : EI+
RT : 1.65 min Scan#: (48,53)
Elements : C 30/0, H 40/0, O 10/0
Mass Tolerance : 1000ppm, [illegible]mmu if m/z < 3, 5mmu if m/z > 5
Unsaturation (U.S.) : -0.5 - 30.0

Observed m/z	Int%	Err[ppm / mmu]	U.S.	Composition
510.2616	33.8	-0.2 / -0.1	12.0	C 30 H 38 O 7

Figure S1. EI/MS data of MRE-23-6-6 from the Root Bark of *Morus alba*.

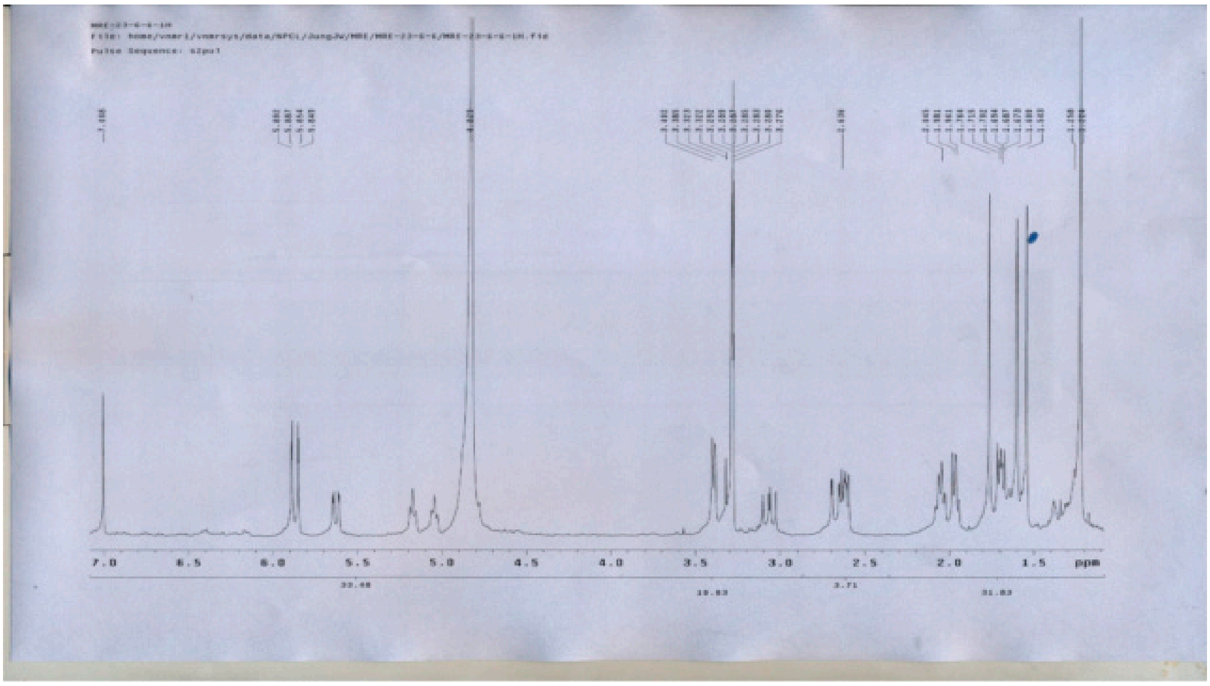

Figure S2. ^{1}H-NMR (400 MHz) spectrum of MRE-23-6-6 from the Root Bark of *Morus alba* (in $CDCl_3$).

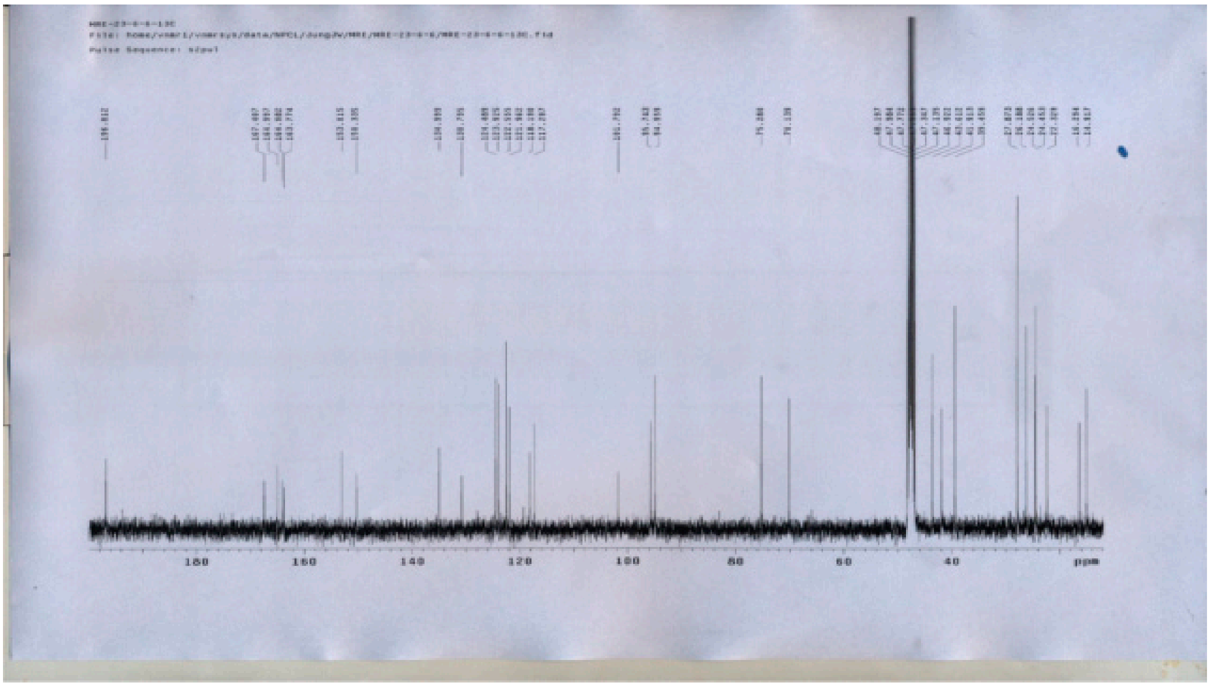

Figure S3. ^{13}C-NMR (100 MHz) spectrum of MRE-23-6-6 from the Root Bark of *Morus alba* (in $CDCl_3$).

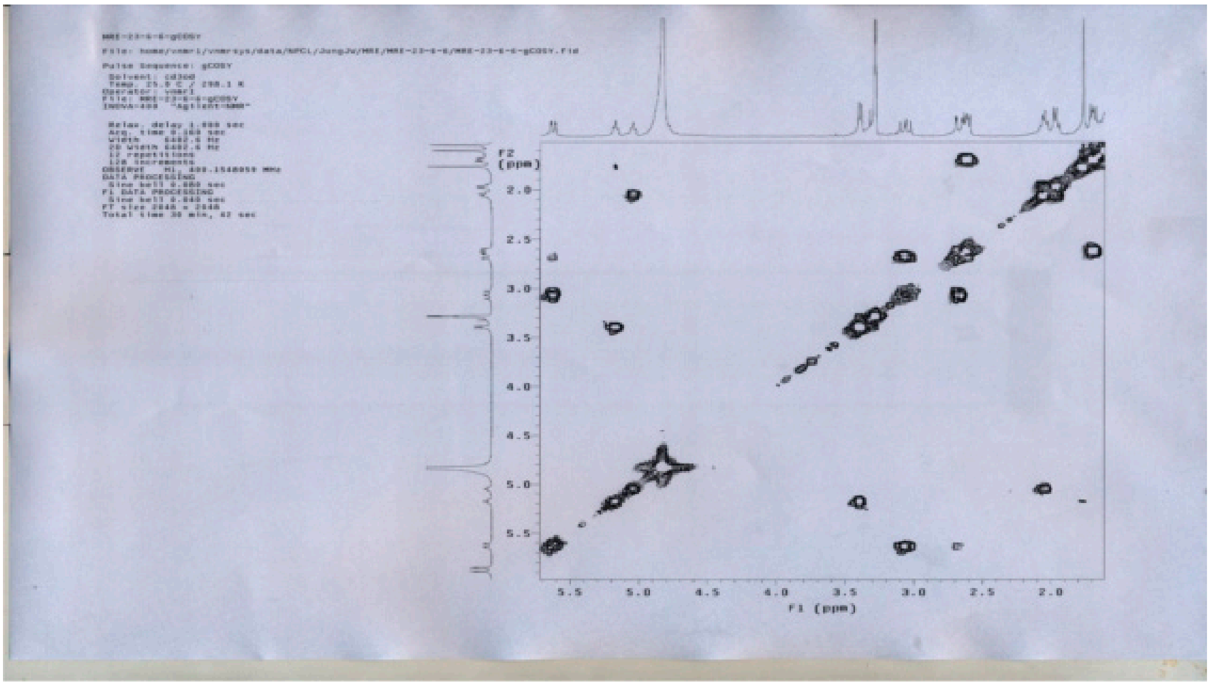

Figure S4. gCOSY spectrum of MRE-23-6-6 from the Root Bark of *Morus alba* (in $CDCl_3$).

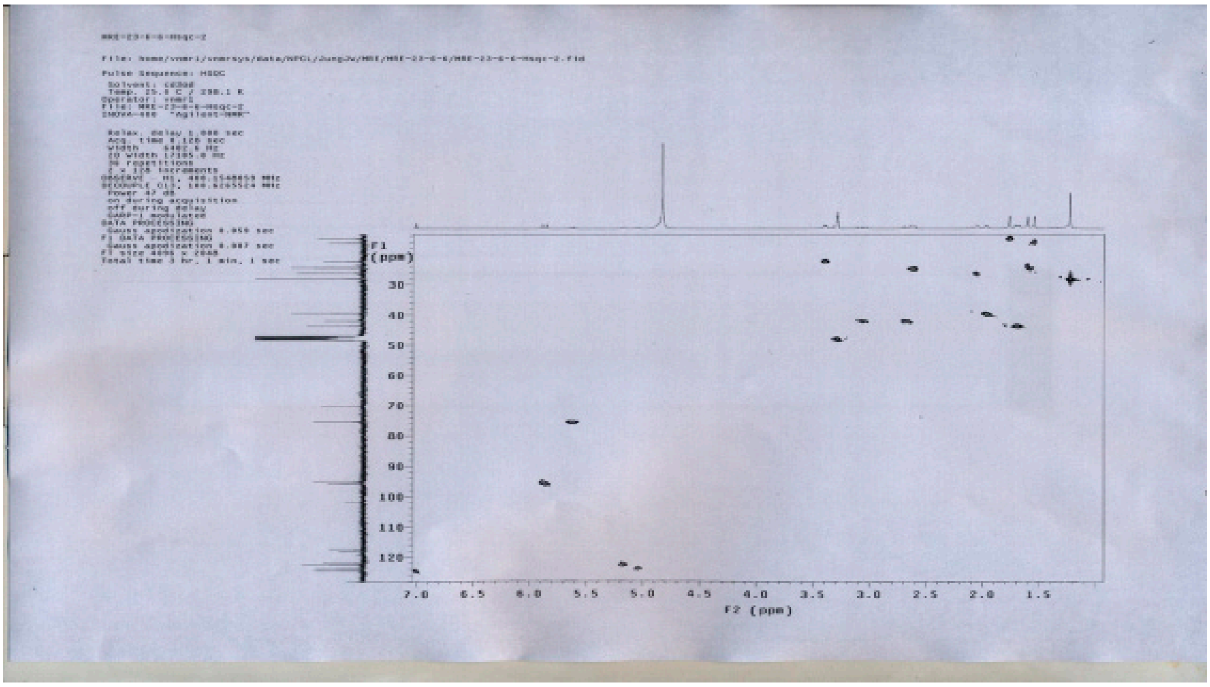

Figure S5. gHSQC spectrum of MRE-23-6-6 from the Root Bark of *Morus alba* (in $CDCl_3$).

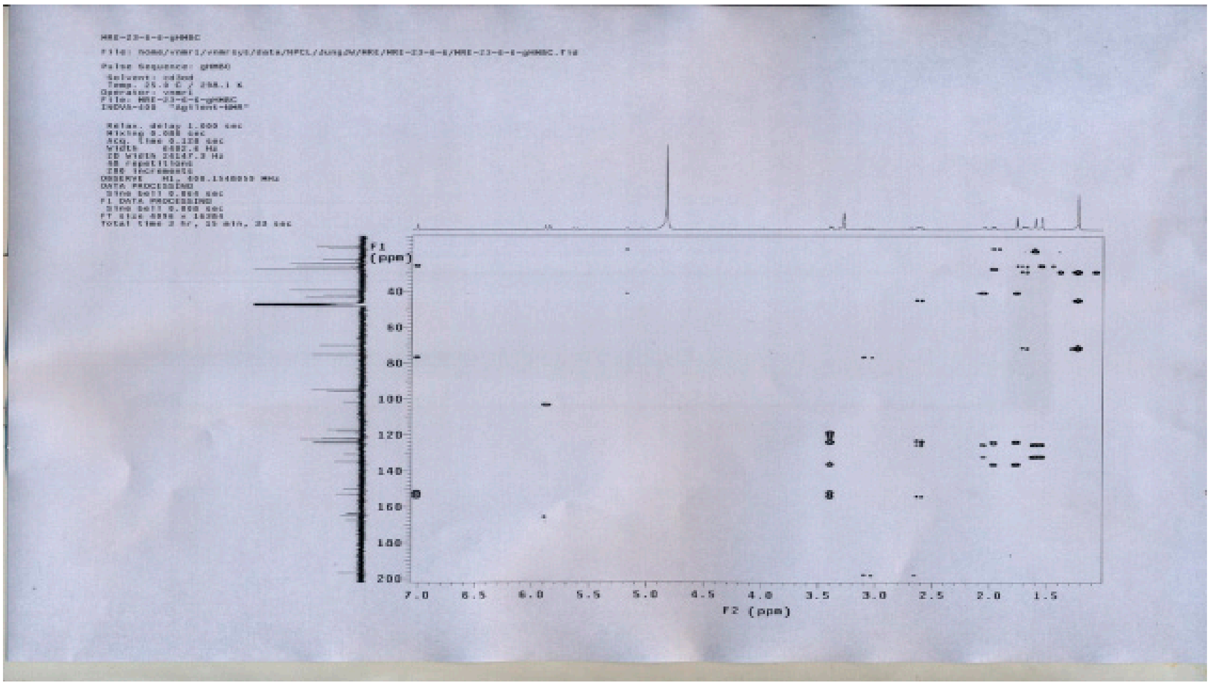

Figure S6. gHMBC spectrum of MRE-23-6-6 from the Root Bark of *Morus alba* (in $CDCl_3$).

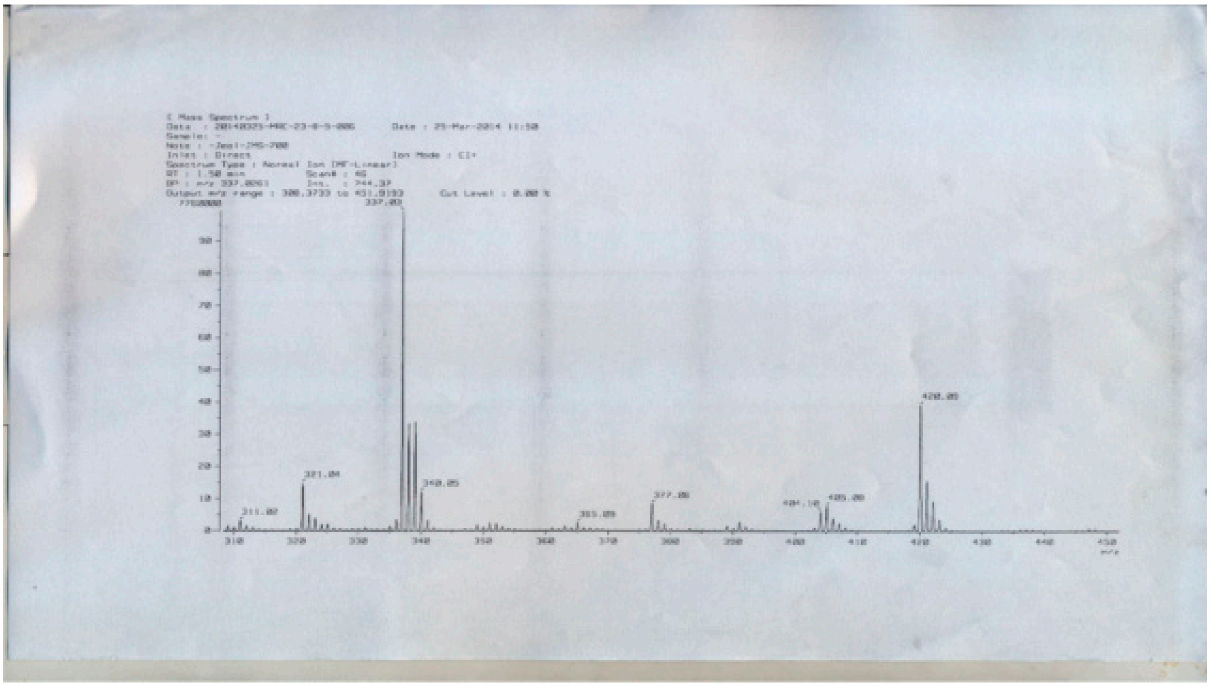

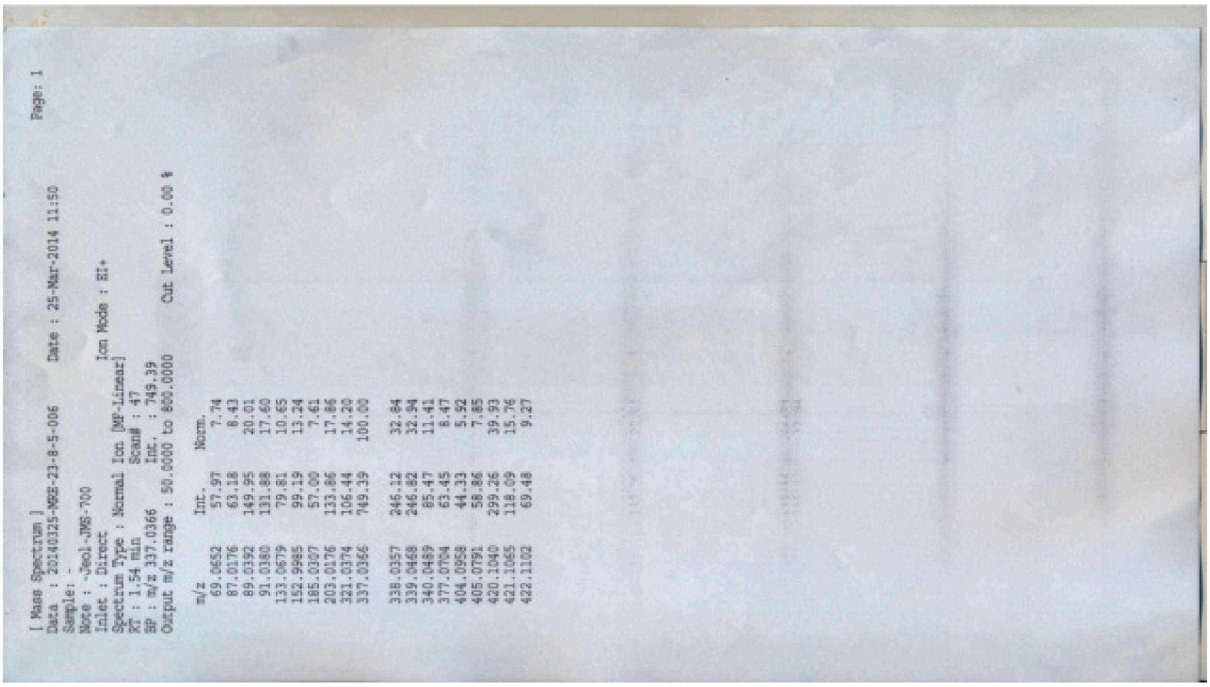

[Mass Spectrum]
Data : 20140325-MRE-23-8-5-006 Date : 25-Mar-2014 11:50 Page: 1
Sample: -
Note : -Jeol-JMS-700
Inlet : Direct Ion Mode : EI+
Spectrum Type : Normal Ion [MF-Linear]
RT : 1.54 min Scan# : 47
BP : m/z 337.0366 Int. : 749.39
Output m/z range : 50.0000 to 800.0000 Cut Level : 0.00 %

m/z	Int.	Norm.
69.0652	57.97	7.74
87.0176	63.18	8.43
89.0392	149.95	20.01
91.0380	131.88	17.60
133.0679	79.81	10.65
152.9985	99.19	13.24
185.0307	57.00	7.61
203.0176	133.86	17.86
321.0374	106.44	14.20
337.0366	749.39	100.00
338.0357	246.12	32.84
339.0468	246.82	32.94
340.0489	85.47	11.41
377.0704	63.45	8.47
404.0958	44.33	5.92
405.0791	58.86	7.85
420.1040	299.26	39.93
421.1065	118.09	15.76
422.1102	69.48	9.27

Figure S7. *Cont.*

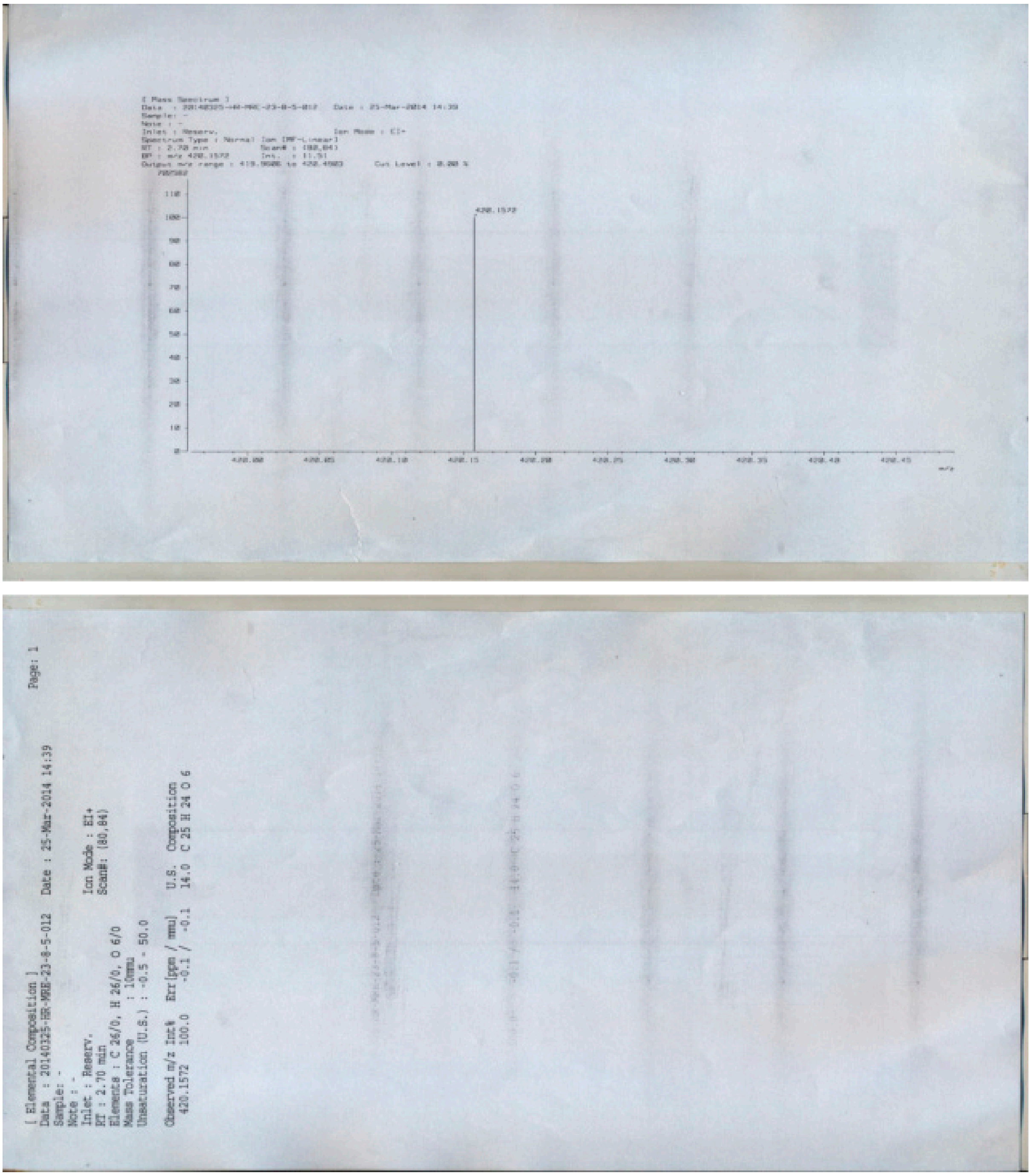

Figure S7. EI/MS data of MRE-23-8-5 from the Root Bark of *Morus alba*.

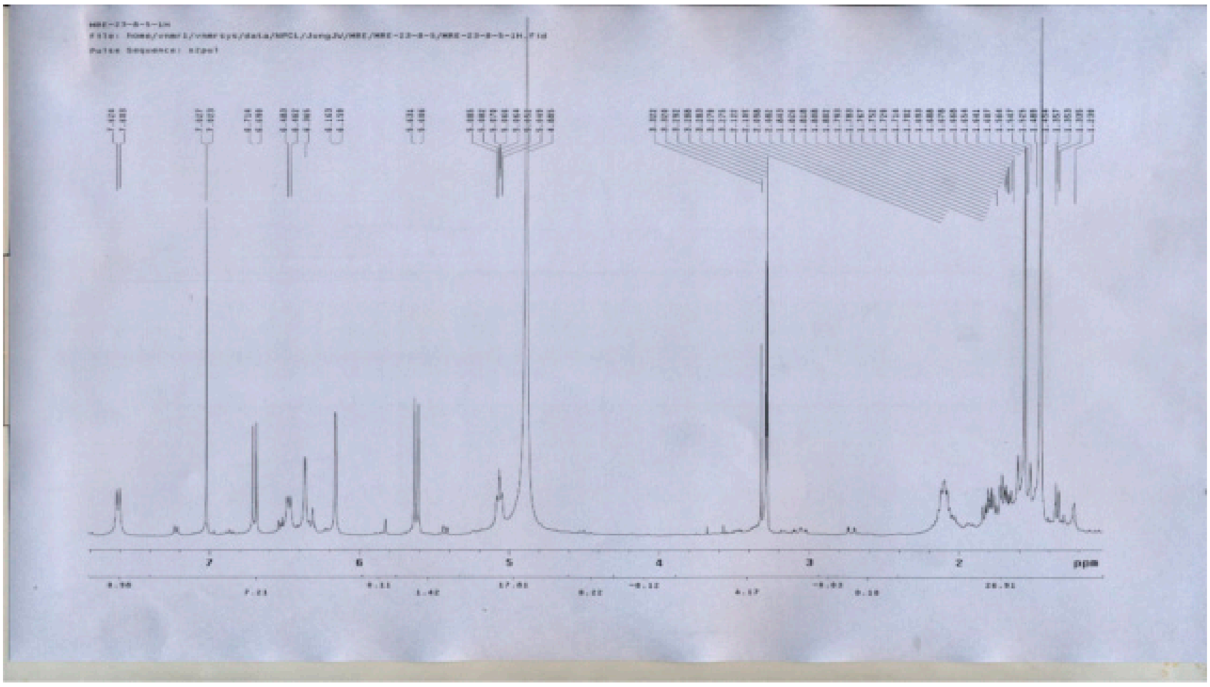

Figure S8. ^{1}H-NMR (400 MHz) spectrum of MRE-23-8-5 from the Root Bark of *Morus alba* (in $CDCl_3$).

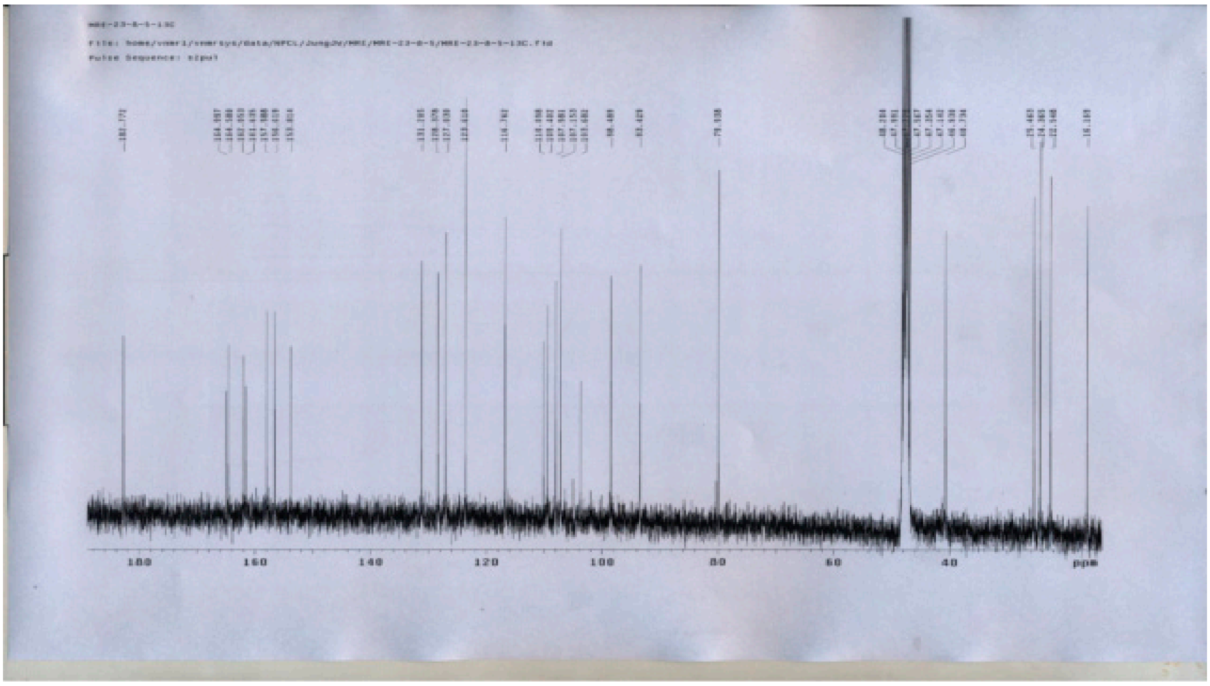

Figure S9. ^{13}C-NMR (100 MHz) spectrum of MRE-23-8-5 from the Root Bark of *Morus alba* (in $CDCl_3$).

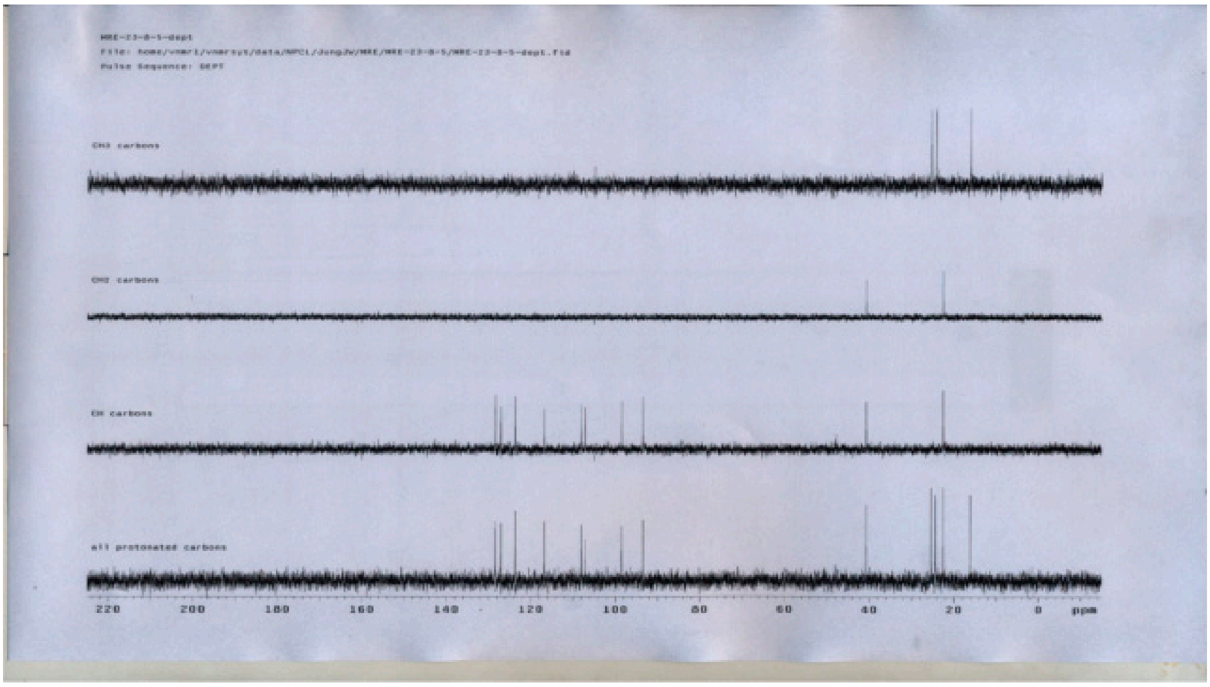

Figure S10. DEPT spectrum of MRE-23-8-5 from the Root Bark of *Morus alba* (in $CDCl_3$).

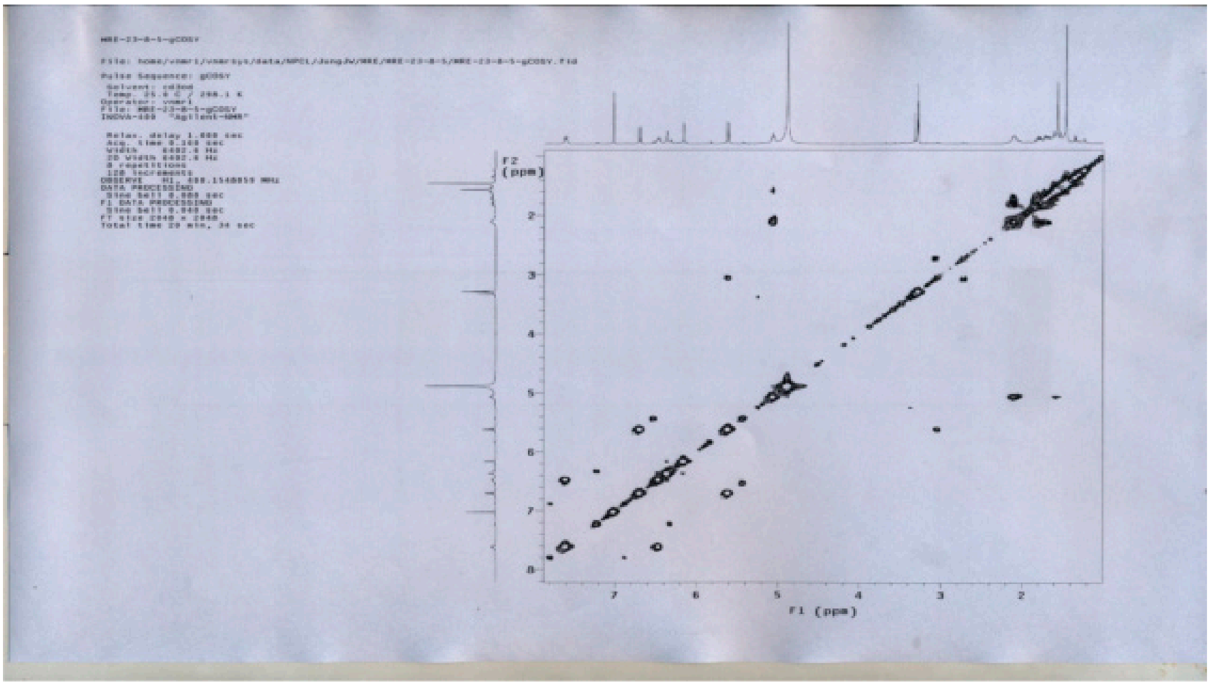

Figure S11. gCOSY spectrum of MRE-23-8-5 from the Root Bark of *Morus alba* (in $CDCl_3$).

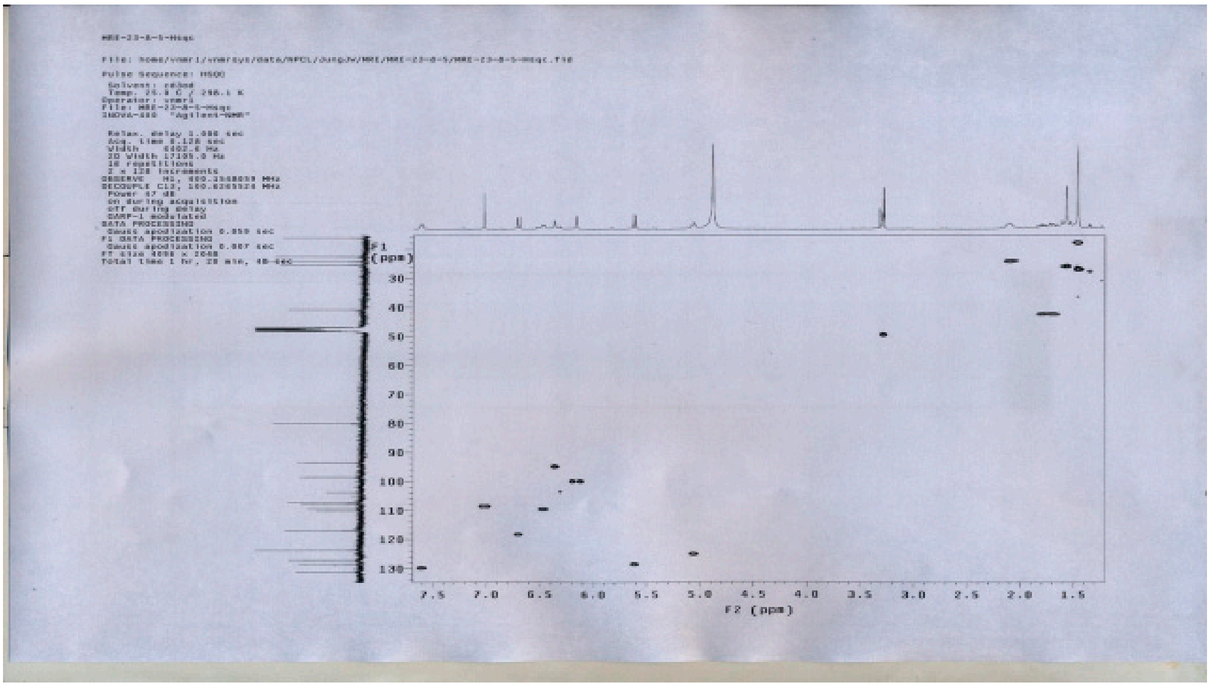

Figure S12. gHSQC spectrum of MRE-23-8-5 from the Root Bark of *Morus alba* (in $CDCl_3$).

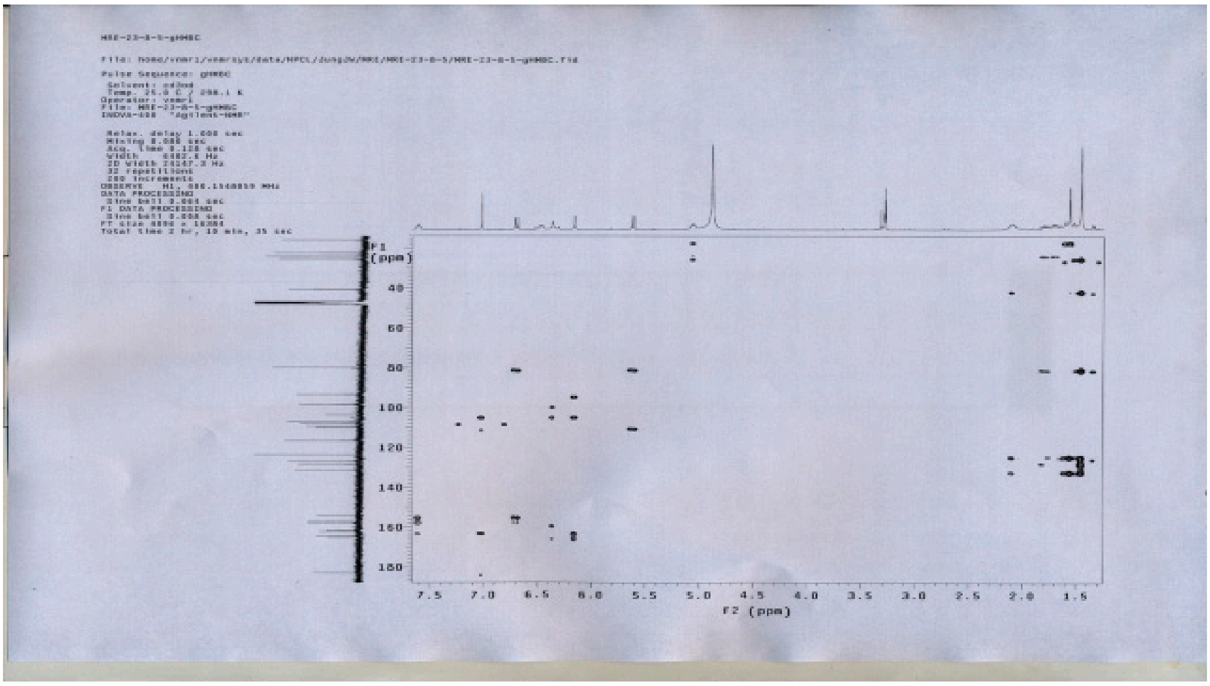

Figure S13. gHMBC spectrum of MRE-23-8-5 from the Root Bark of *Morus alba* (in $CDCl_3$).

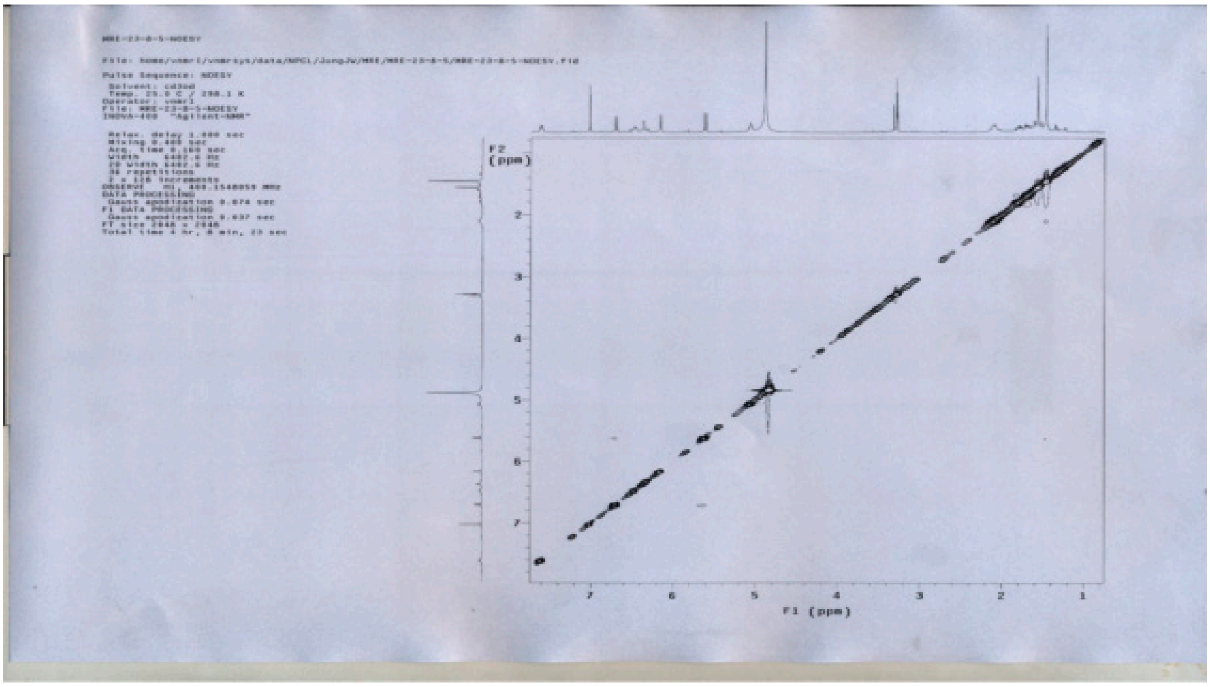

Figure S14. NOESY spectrum of MRE-23-8-5 from the Root Bark of *Morus alba* (in $CDCl_3$).

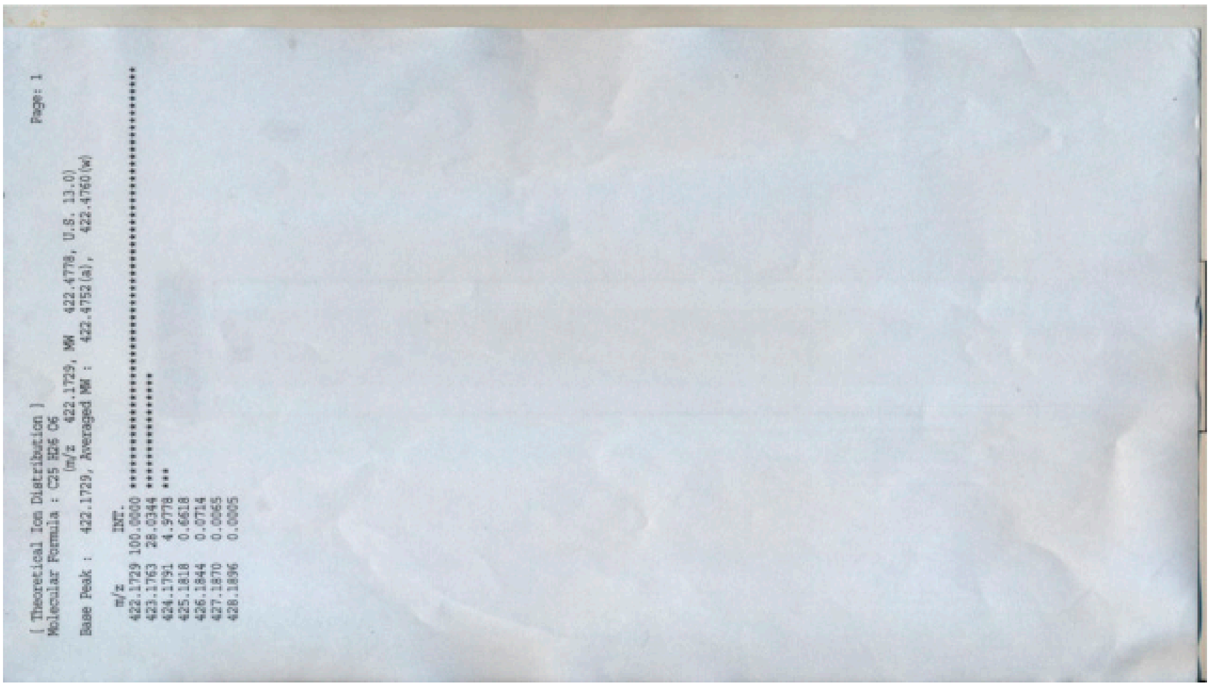

Figure S15. *Cont.*

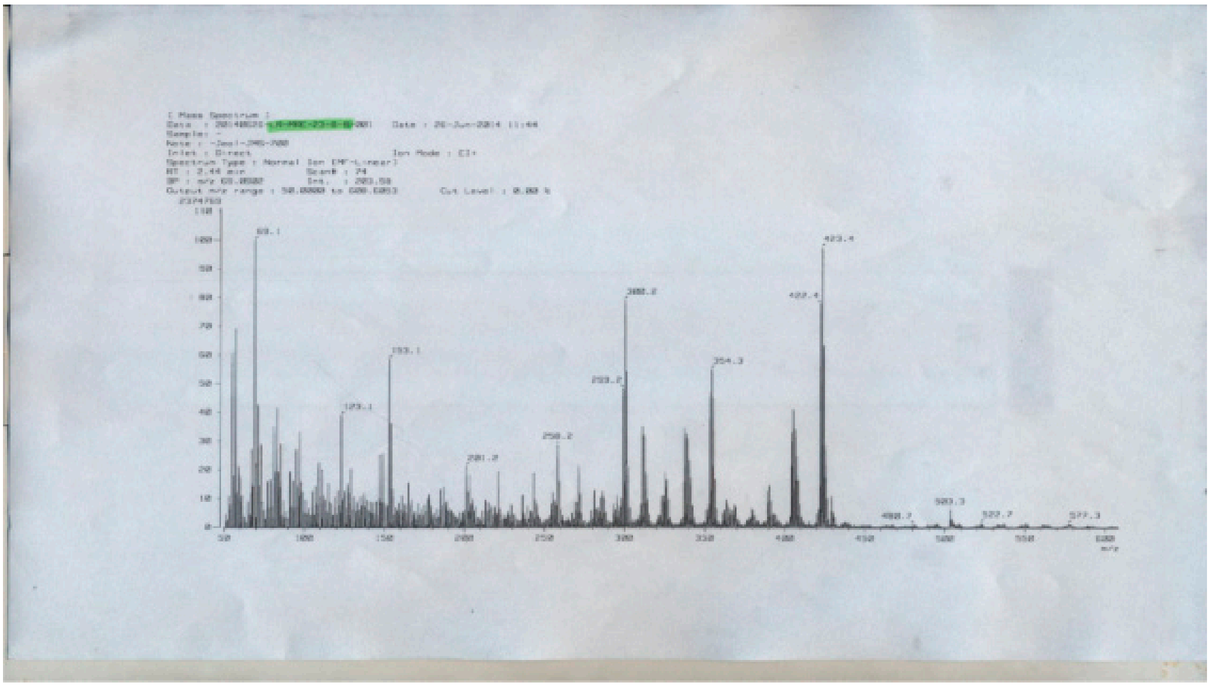

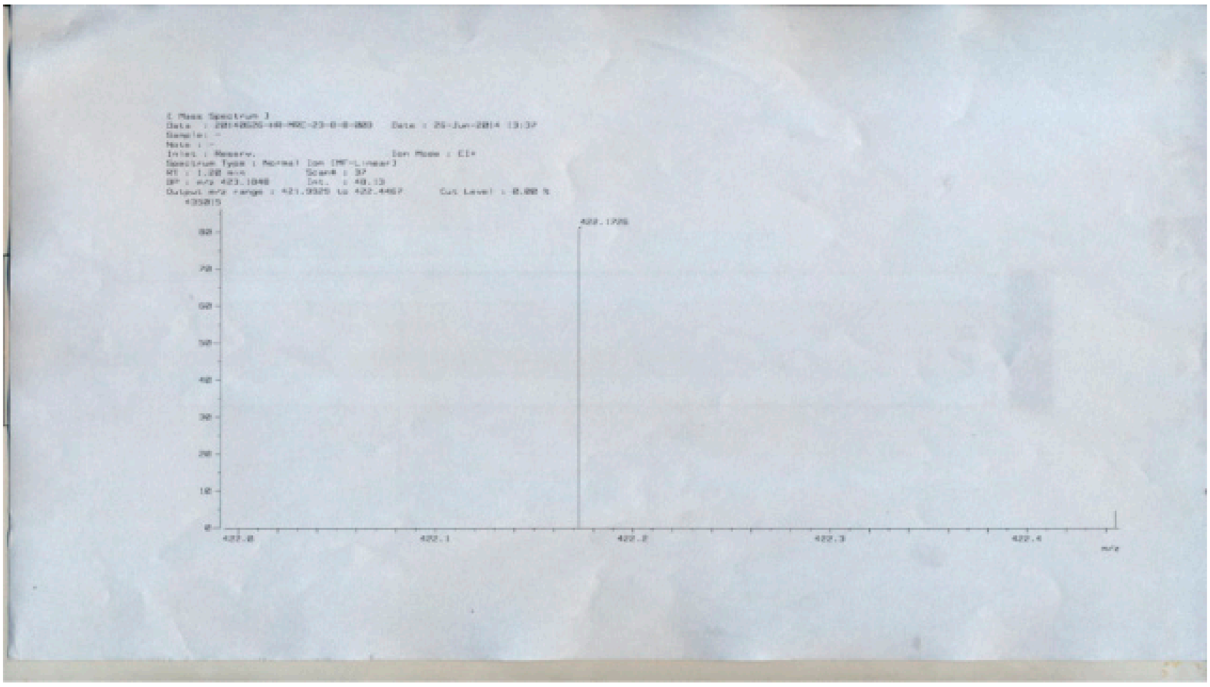

Figure S15. *Cont.*

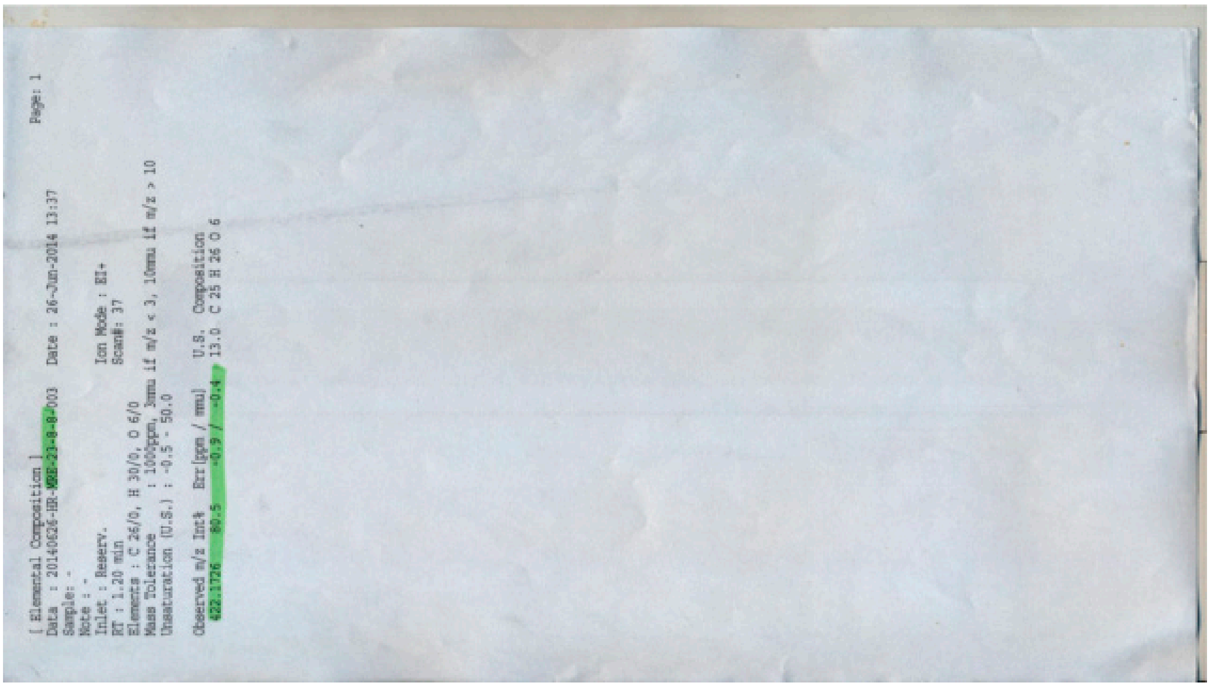
[Elemental Composition] Page: 1
Data : 20140626-HR-MRE-23-8-8-003 Date : 26-Jun-2014 13:37
Sample: -
Note : -
Inlet : Reserv. Ion Mode : EI+
RT : 1.20 min Scan#: 37
Elements : C 26/0, H 30/0, O 6/0
Mass Tolerance : 1000ppm, 3mmu if m/z < 3, 10mmu if m/z > 10
Unsaturation (U.S.) : -0.5 - 50.0

Observed m/z	Int%	Err[ppm / mmu]	U.S.	Composition
422.1726	80.5	-0.9 / -0.4	13.0	C 25 H 26 O 6

Figure S15. EI/MS data of MRE-23-8-8 from the Root Bark of *Morus alba*.

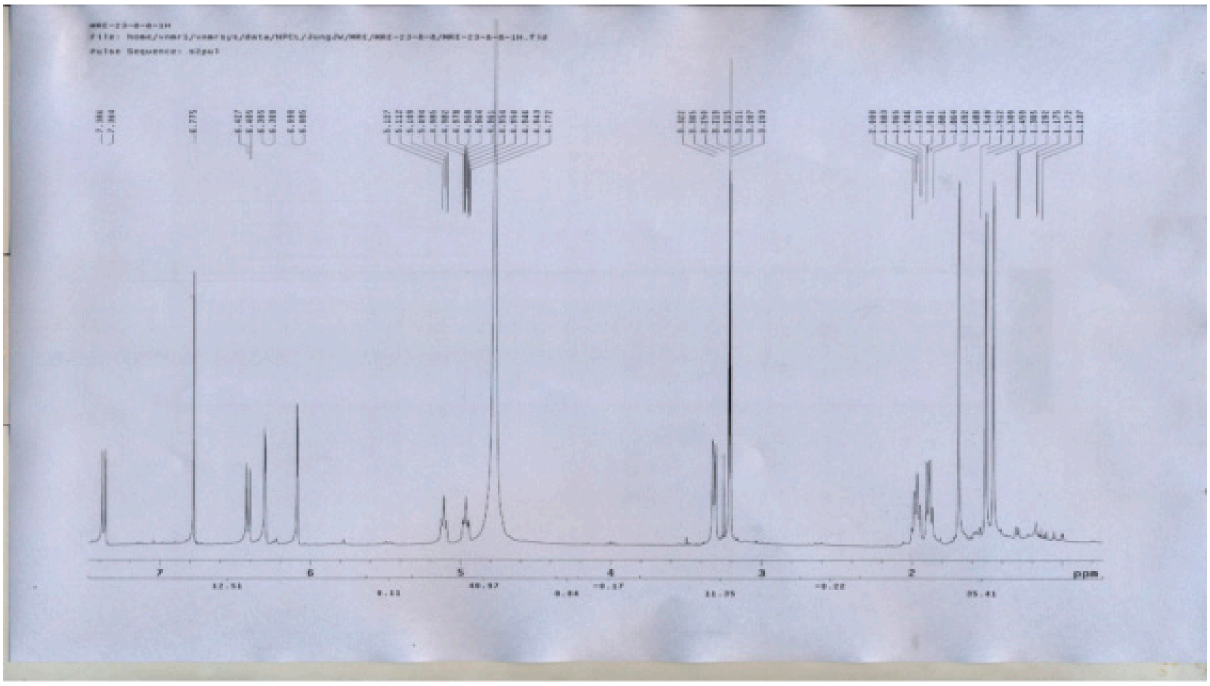

Figure S16. ^{1}H-NMR (400 MHz) spectrum of MRE-23-8-8 from the Root Bark of *Morus alba* (in $CDCl_3$).

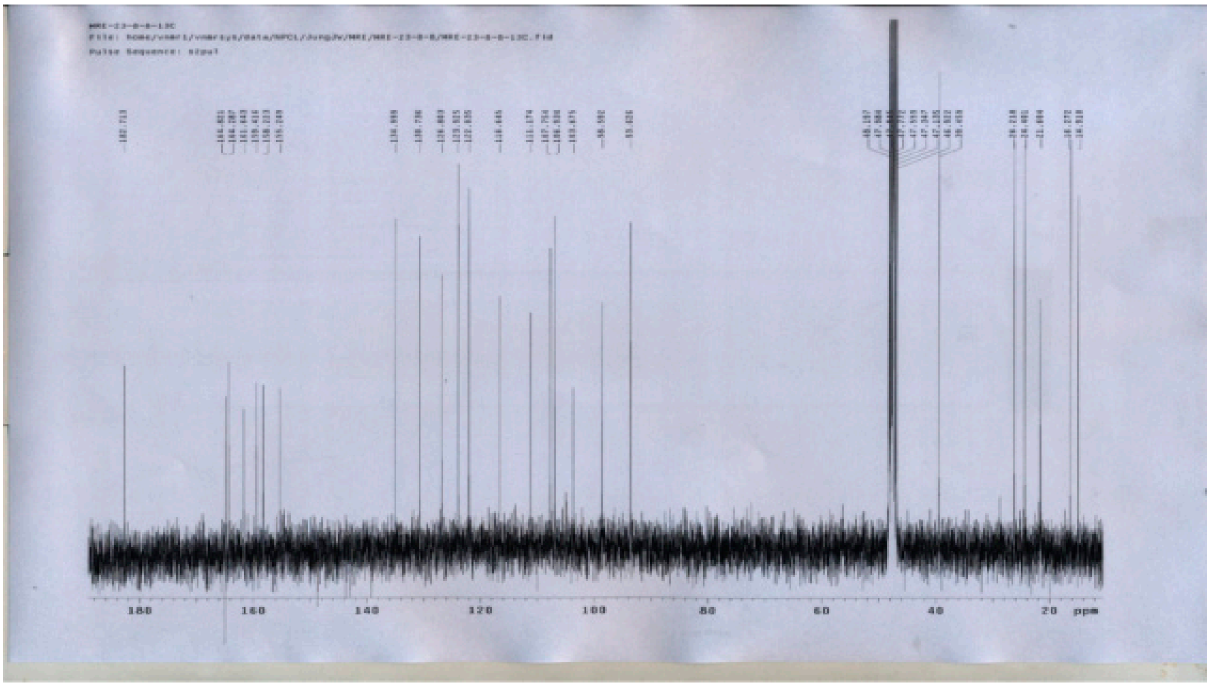

Figure S17. ^{13}C-NMR (100 MHz) spectrum of MRE-23-8-8 from the Root Bark of *Morus alba* (in $CDCl_3$).

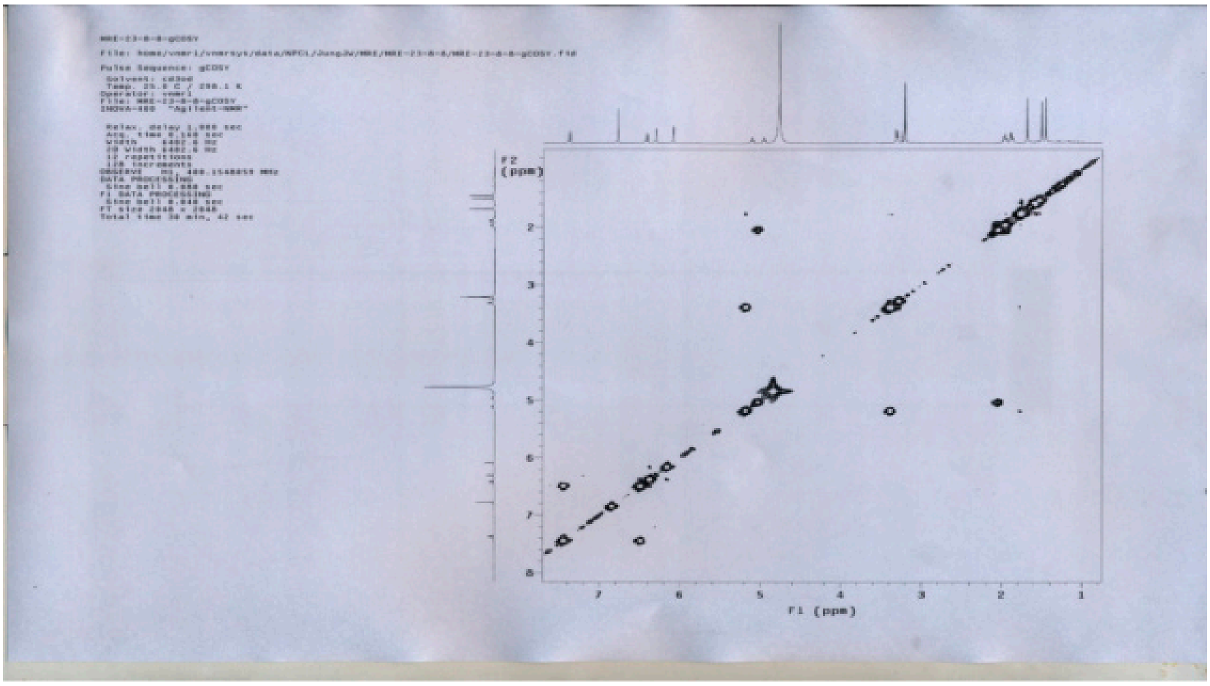

Figure S18. gCOSY spectrum of MRE-23-8-8 from the Root Bark of *Morus alba* (in $CDCl_3$).

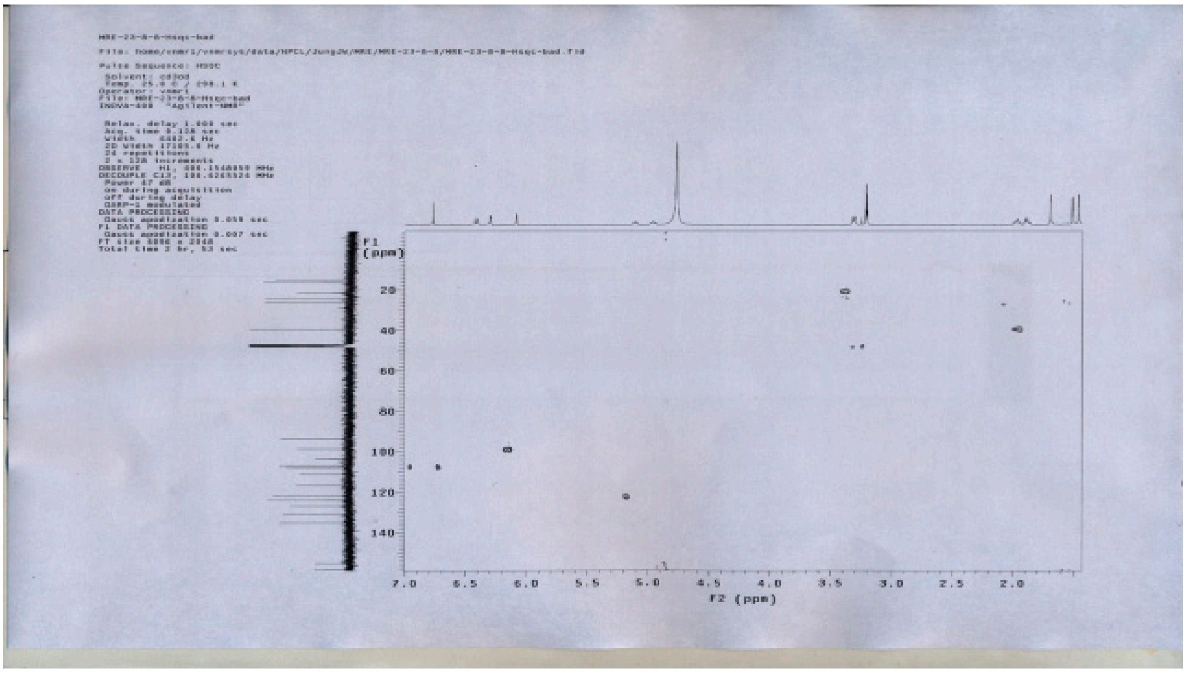

Figure S19. gHSQC spectrum of MRE-23-8-8 from the Root Bark of *Morus alba* (in $CDCl_3$).

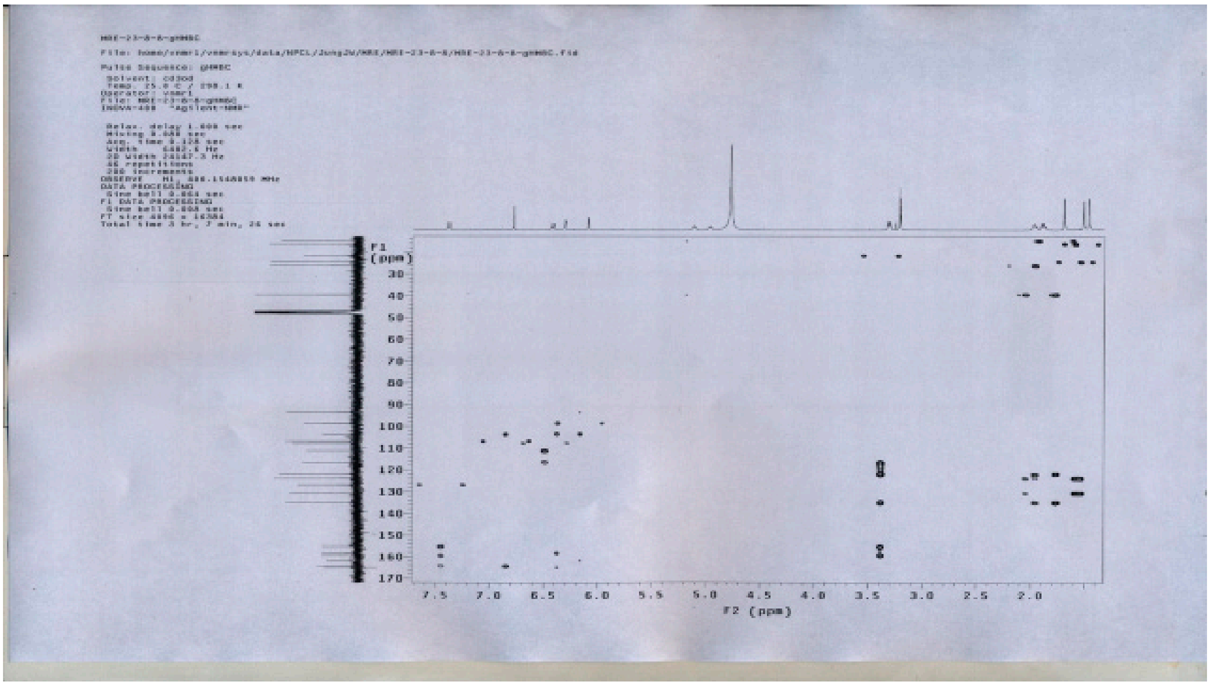

Figure S20. gHMBC spectrum of MRE-23-8-8 from the Root Bark of *Morus alba* (in $CDCl_3$).
